# Supplementary material for: Disentangling the link between maternal influences on birth weight and disease risk in 36,211 genotyped mother–child pairs
Source: Commun Biol. 2024 Feb 12;7:175. doi: 10.1038/s42003-024-05872-9 (PMC10861556; doi:10.1038/s42003-024-05872-9)
Supplement: Supplementary file 2 — Supplementary Information [file 42003_2024_5872_MOESM2_ESM.pdf]

Supplementary Information

Supplementary Fig. 1: Schematic illustration of relationships between the studied PGSs and their correlations.

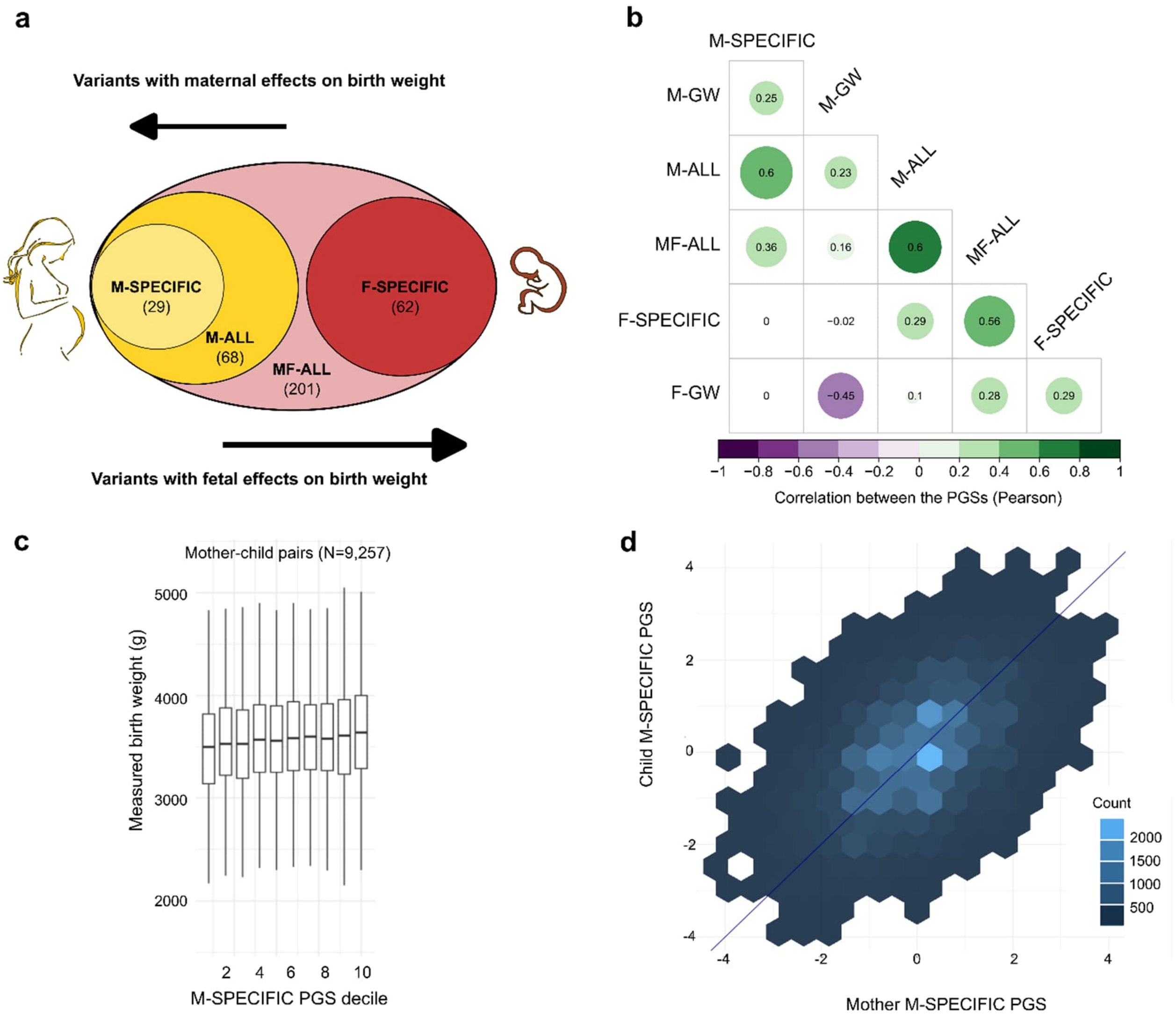

Panel a illustrates relationships between unweighted lead-SNP based PGSs (M-SPECIFIC, M-ALL, MF-ALL, and F-SPECIFIC), partly overlapping in the depicted manner. Yellow and red shades indicate PGSs reflecting mostly maternal or fetal contributions on birth weight, respectively. M-SPECIFIC is solely based on variants with maternal effects on birth weight, M-ALL is based on variants with maternal effects, some of which have also fetal effects, MF-ALL is mostly based on SNPs acting through the child's genome to affect birth weight, although it also contains maternal SNPs, and F-SPECIFIC is based on SNPs with exclusive fetal effects on birth weight. Numbers in parentheses refer to the number of variants in each score. Panel b shows the Pearson correlations between the PGSs, including the genome-wide scores for maternal and fetal effects on birth weight (M-GW and F-GW). Green shades indicate positive, and purple shades indicate negative correlations. Panel

c demonstrates the effect of mother's M-SPECIFIC PGS on child's birth weight in mother-child pairs (N=9,257 children with birth weight data). Panel d illustrates the correlation (Pearson r = 0.504) between the mother's and child's standardised M-SPECIFIC PGS scores in the FinnGen mother-child pairs. Each hexagon is based on data from at least 15 mother-child pairs. Lighter blue shades indicate more mother-child pairs under each hexagonal bin.

Supplementary Fig. 2: Simulations showing how the PGS associations could look like under four different scenarios.

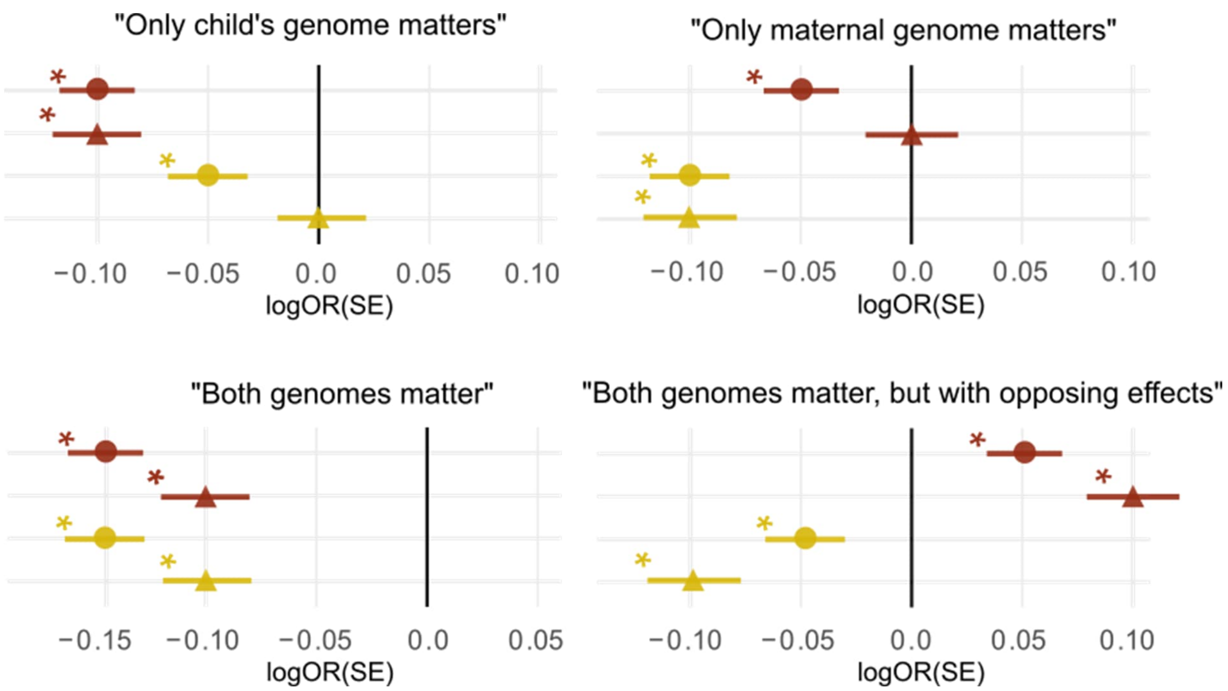

The scenarios are (1) only child genome matters, 2) only maternal genome matters, 3) both genomes matter and 4) both genomes matter, exerting opposite effects. The simulations are based on true effect size of logOR -0.1 and N cases =4,700. Red = effect estimates for the child's PGS. Yellow = effect estimates for the mother's PGS. Round dots = effect from a model where only mother's or child's PGS has been included into the analysis (unadjusted). Triangles = effect from a model where both mother's and child's PGS have been included into the same analysis (adjusted). The error bars for the point estimates denote standard error (SE). * *P*<0.05.

Supplementary Fig. 3: Associations of the six birth weight PGSs with disease risk in the children from mother-child pairs.

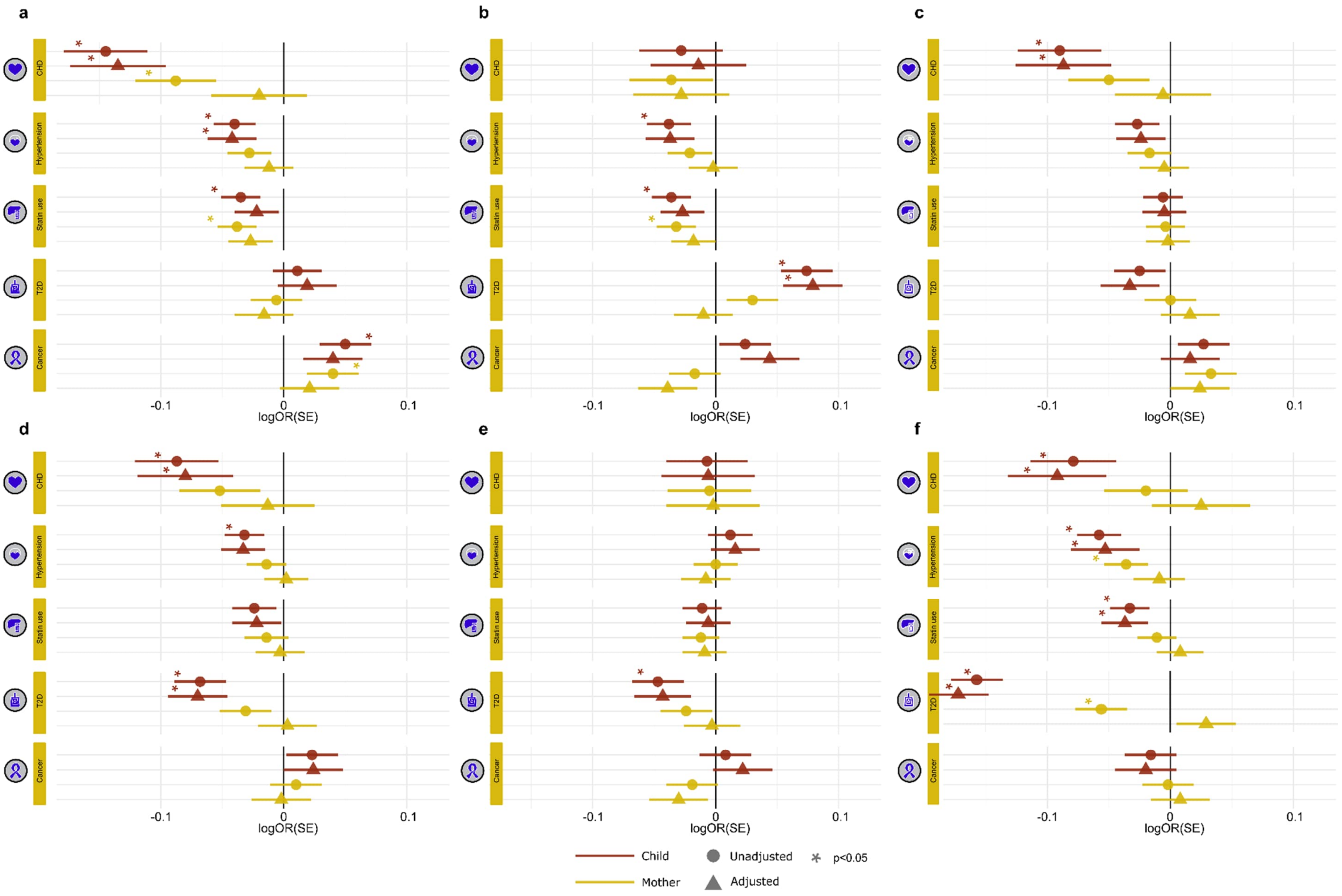

The figure illustrates the association between the child's own PGS (red) and the mother's PGS (yellow) with the child's disease risk in mother-child pairs. a Effect of 1SD increase in M-SPECIFIC on disease risk. b Effect of 1SD increase in M-GW on disease risk. c Effect of 1SD increase in M-ALL on disease risk. d Effect of 1SD increase in MF-ALL on disease risk. e Effect of 1SD increase in F-SPECIFIC on disease risk. f Effect of 1SD increase in F-GW on disease risk. Round dots illustrate cases when only one PGS is used in the analyses (unadjusted), and triangles indicate analyses including PGSs from both mothers and their children (adjusted). The error bars denote standard error (SE). * Denotes a statistically significant association between a PGS and disease risk in children ($P<0.05$). Coronary heart disease (CHD) N=996; Hypertension N=4,738; Statin use N=6,150; type 2 diabetes (T2D) N=2,841; Cancer N=2,790.

Supplementary Fig. 4: Change in effect sizes between different models.

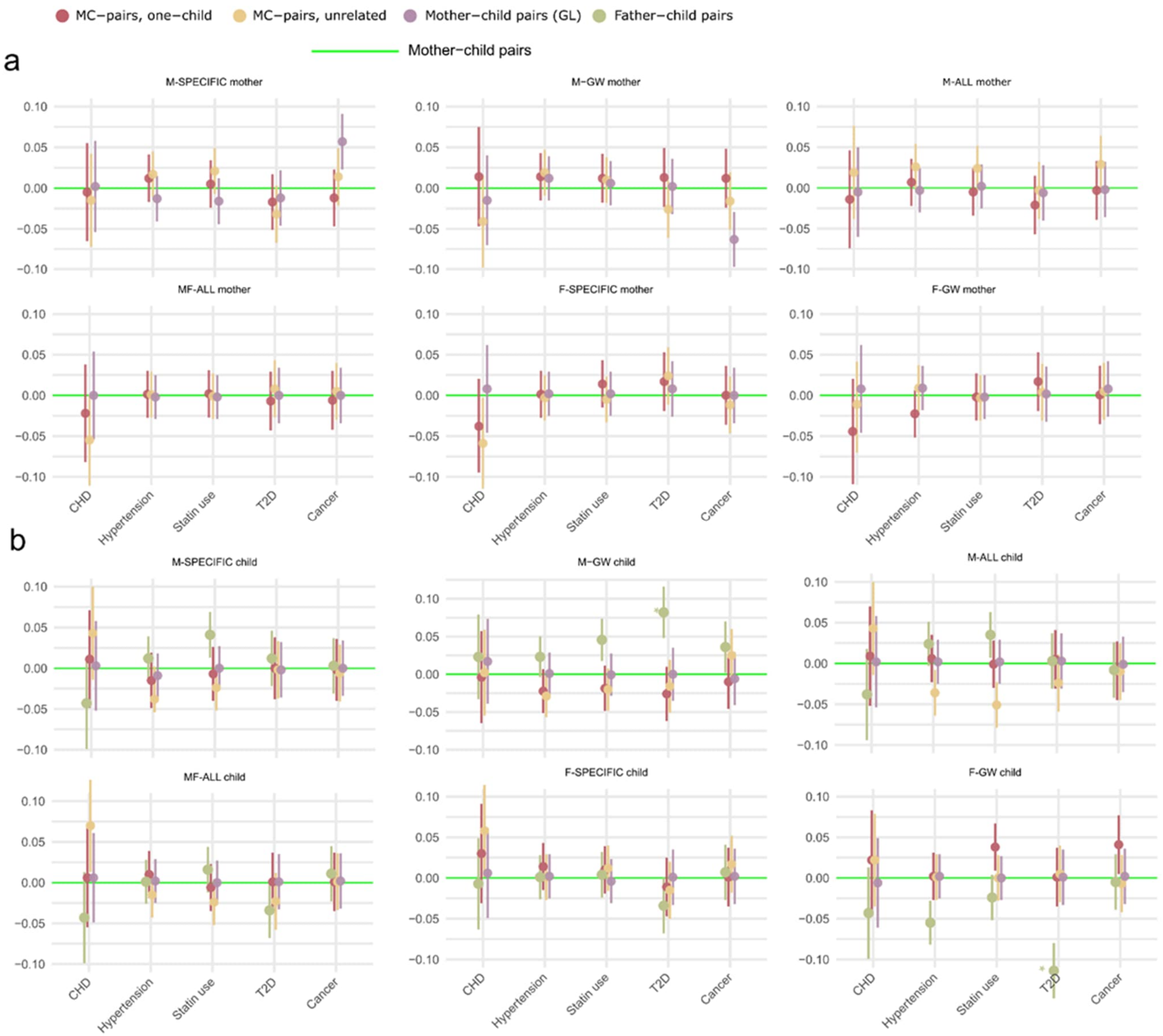

a Change in the estimates for mother's PGS effect size between the main and sensitivity analyses. b Change in the estimates for child PGS effect size between them main and sensitivity analyses. Units are logOR. Dots indicate point estimates with lines showing error bars (SE). Bright green line refers to effect in the main analyses in mother-child pairs (mother and child PGS effect on disease taking into account both PGSs). Red=effect difference in mother-child pairs where only one child has been included, yellow= effect difference in unrelated pairs, lilac= effect difference in mother-child pairs adjusting for PGS for maternal effects on gestational length (GL), olive green = the effect difference for children in the father-child pairs. The calculations are based on Supplementary Data 4-6. Error bars denote standard error (SE) for difference between the baseline model (bright green) and sensitivity analyses. The asterisk marks statistically significant difference in effect sizes between the two models ($P<0.05$).

Supplementary Fig. 5: Associations of the six birth weight PGSs with disease risk in the children from father-child pairs.

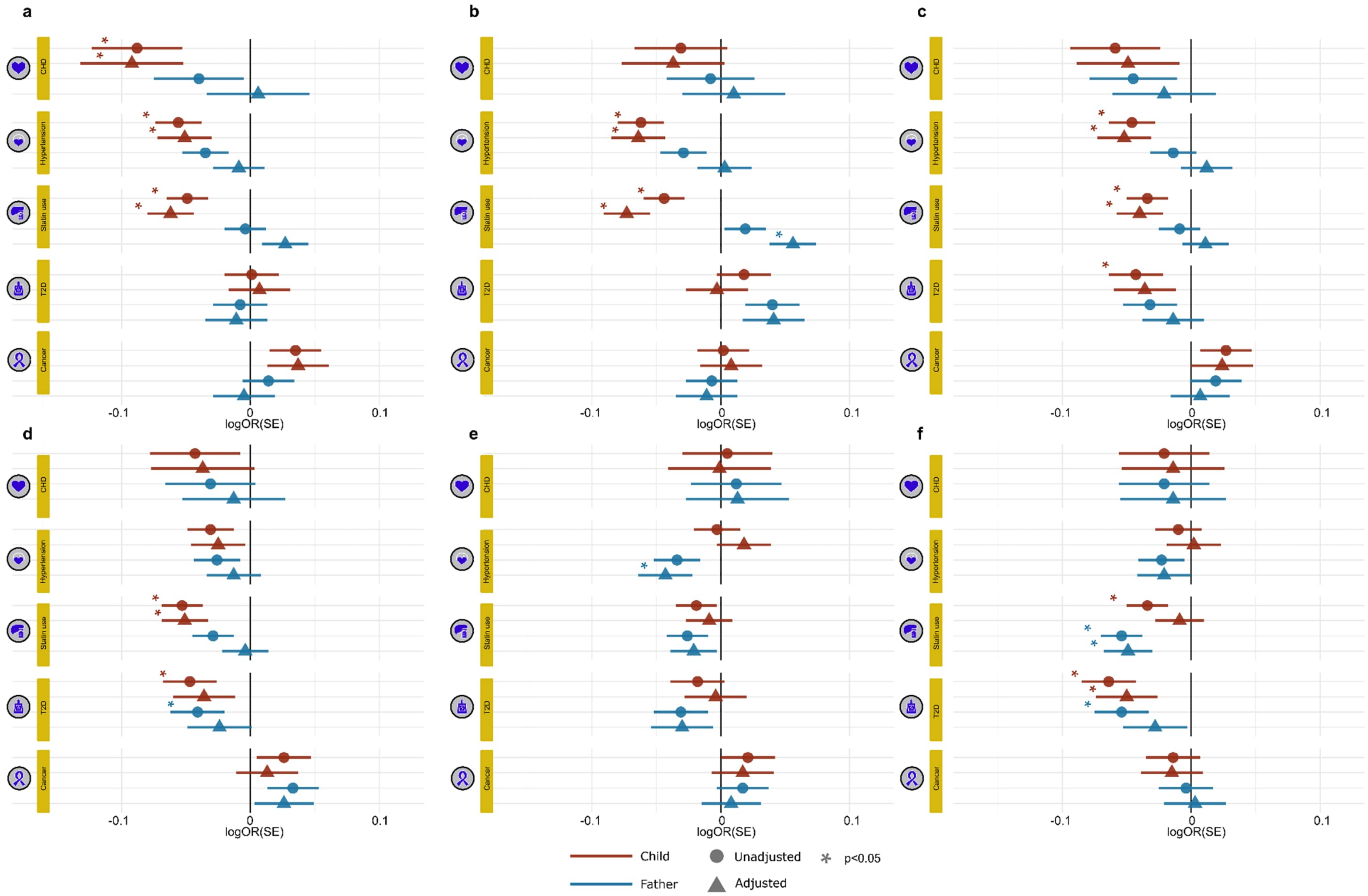

The figure shows how the child's own PGS (red) and father's PGS (blue) associate with child's disease risk in the father-child pairs. a Effect of 1SD increase in M-SPECIFIC on disease risk. b Effect of 1SD increase in M-GW on disease risk. c Effect of 1SD increase in M-ALL on disease risk. d Effect of 1SD increase in MF-ALL on disease risk. e Effect of 1SD increase in F-SPECIFIC on disease risk. f Effect of 1SD increase in F-GW on disease risk. Round dots illustrate cases when only one PGS is used in the analyses (unadjusted), and triangles indicate that the analyses include PGSs from both fathers and their children (adjusted). The error bars denote standard error (SE). * Denotes a statistically significant association between a PGS and disease risk in children ($P<0.05$). Coronary heart disease (CHD) N=992; Hypertension N=4,631; Statin use N=6,118; type 2 diabetes (T2D) N=2,678; Cancer N=2,966.

Supplementary Fig. 6: Association of PGSs with disease in sex matched – disease discordant full siblings from FinnGen.

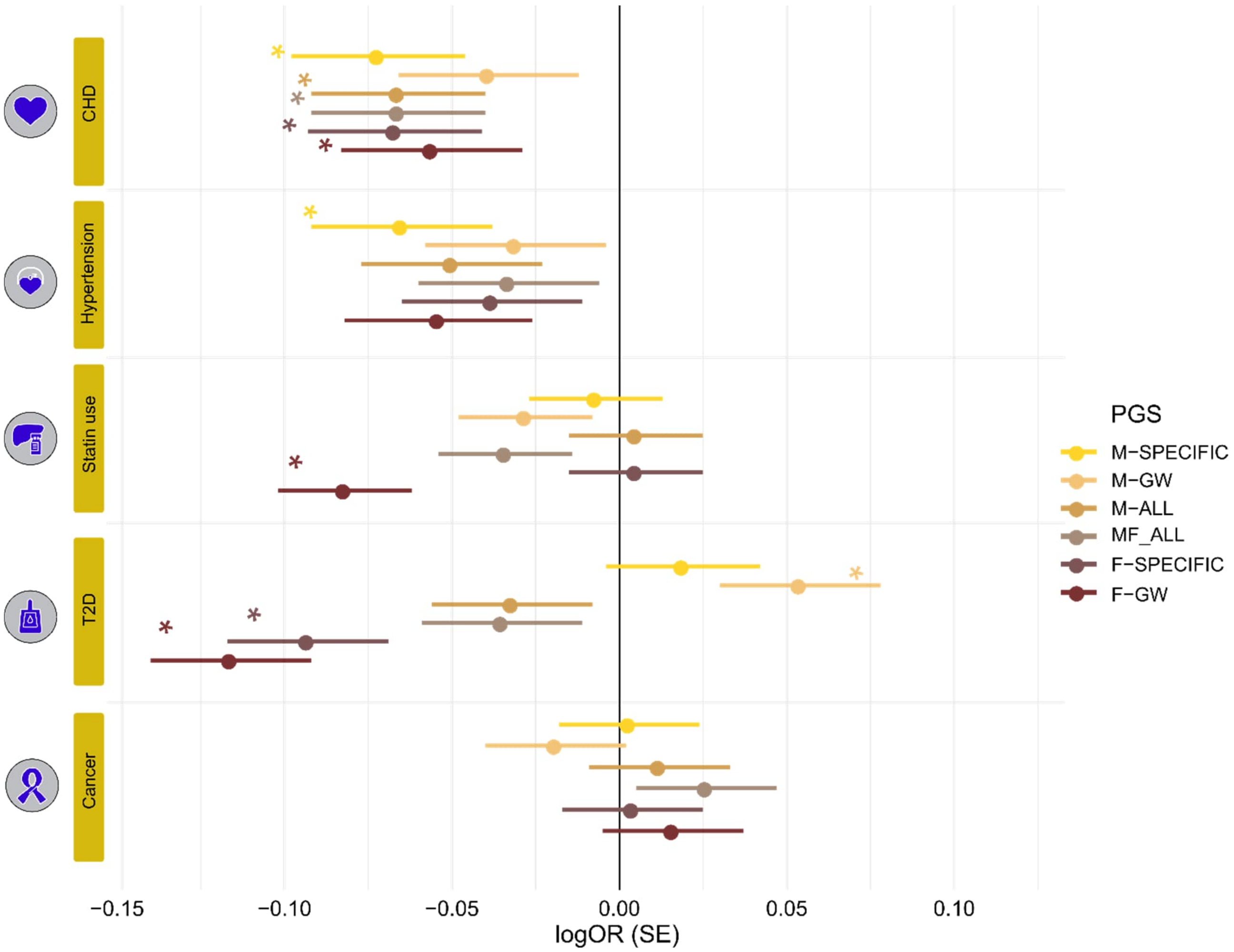

The figure shows the effect of 1SD increase in a birth weight PGS, corresponding to larger birth weight, on disease risk in FinnGen R10 sibling pairs. Colours reflect maternal and fetal PGSs as follows: Yellow=M-SPECIFIC; gold=M-GW; amber=M-ALL; grey=MF-ALL; burgundy=F-SPECIFIC; crimson=F-GW. The error bars denote standard error (SE). * $P<0.05$. The number of cases in the disease discordant sibling pairs: Coronary heart disease (CHD) N=6,016; Hypertension N=6,164; Statin use N=11,505; type 2 diabetes (T2D) N=7,298; Cancer N=9,484.

Supplementary Fig. 7: Illustration of power to detect maternal effects on child disease depending on disease case numbers in the mother-child pairs.

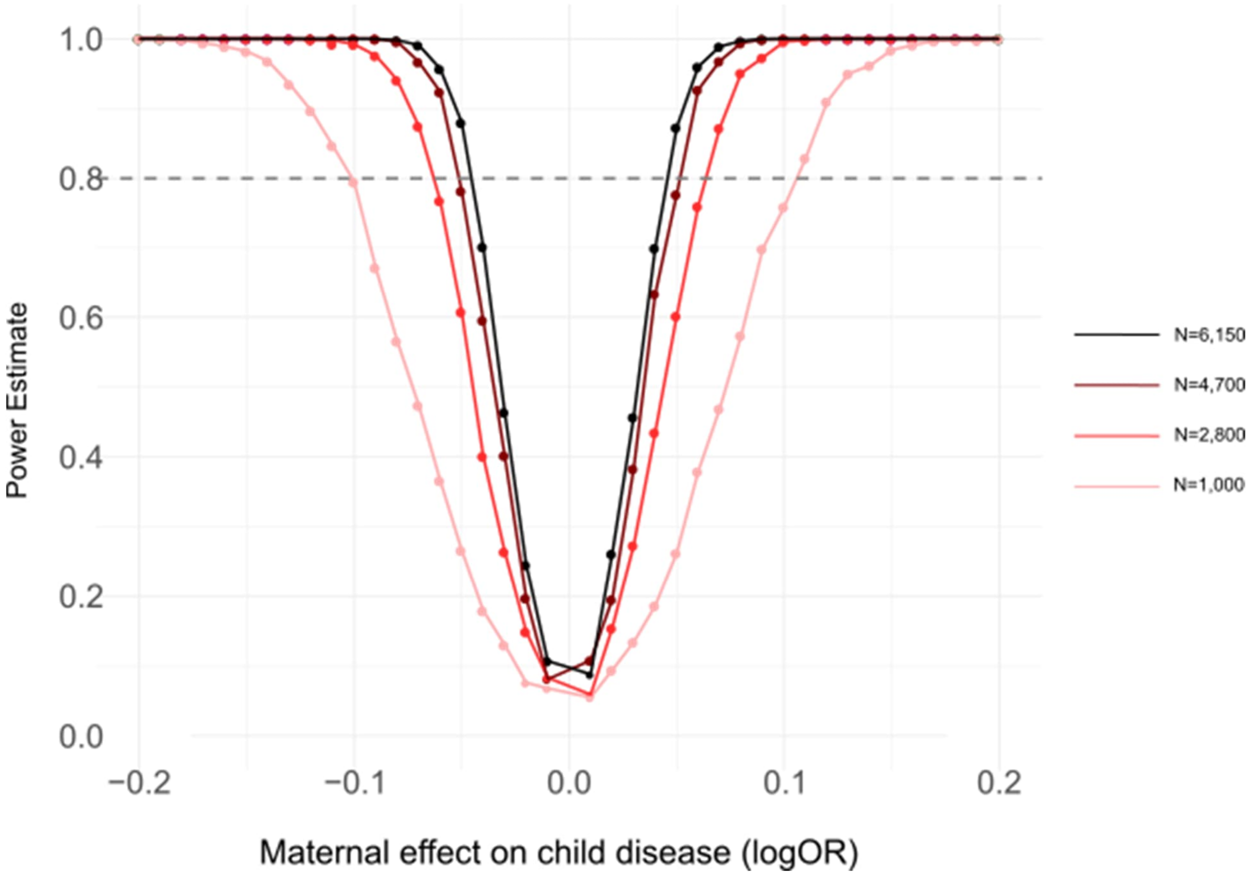

The point estimates and fitted curves illustrate power when taking into account both child and mother effects simultaneously (Supplementary Data 3). The estimates are based on assumed true maternal effects of logOR -0.2 to 0.2 in 0.01 unit intervals in mothers, and no effects in children. Each data point is based on 1000 simulations. Colours indicate number of disease cases in each simulation in 36,211 mother-child pairs. Black = N cases 6,150, dark red N = 4,700, red N =2,800, pink N = 1000, corresponding to observed disease cases in FinnGen mother-child pairs for statin use, hypertension, T2D and cancer, and CHD, respectively.

Supplementary Fig. 8: Cohort demographics.

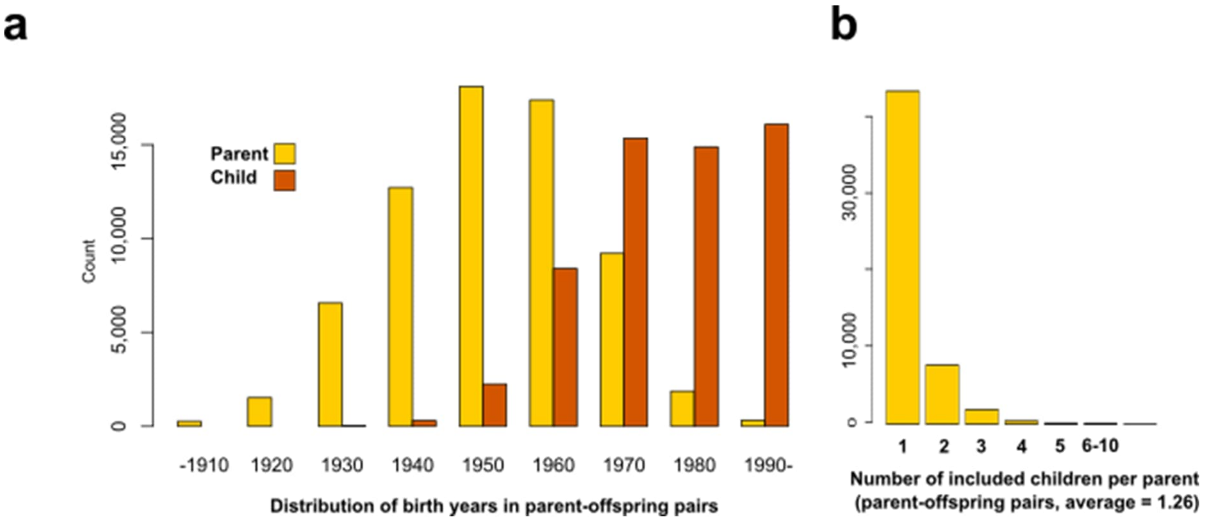

a Distribution of the birth years of the parent-offspring pairs (N=67,986) in FinnGen R10. b Number of children per parent in the dataset.

Supplementary Software 1. Simulate power for binary endpoints in the mother-child pairs.

```
sim_pgs_power <- function(Nmc,Ncases,betao,betam){

library(mvtnorm)

### Simulate mother and child polygenic scores (PGS)

# Covariance matrix for maternal and offspring PGS (r=0.5) scaled to unit variance

sigma <- matrix(c(1,0.5,0.5,1),nrow=2,byrow=T)

# Simulate PGS distributions

mc_pgs <- rmvnorm(Nmc,mean=c(0,0),sigma,method="eigen")

### Construct phenotype

# Set alpha and risk function

k = Ncases/Nmc

alpha = log(k/(1-k))

risk <- exp(alpha+betao*mc_pgs[,1]+betam*mc_pgs[,2])/(1+exp(alpha+betao*mc_pgs[,1]+betam*mc_pgs[,2]))

### Test power

# Case-control vector

cco <- rbinom(Nmc,size=1,risk)

# Generalised linear regression: case status ~ offspring PGS + maternal PGS

out <- summary(glm(cco~mc_pgs[,1]+mc_pgs[,2],family='binomial'))$coefficients

# Output results: intercept, offspring beta, maternal beta, offspring p-value, maternal p-value

sim_res <- data.frame(alpha=out[1,1],betao=out[2,1],betam=out[3,1],pbetao=out[2,4],pbetam=out[3,4])

return(sim_res)

}
```

Supplementary Information

FinnGen consortium members

Full Name	Affiliation	Role1	Role 2
Aarno Palotie	Institute for Molecular Medicine Finland (FIMM), HiLIFE, University of Helsinki, Helsinki; Broad Institute of MIT and Harvard; Massachusetts General Hospital, Boston MA, United States	Steering Committee	Steering Committee
Mark Daly	Institute for Molecular Medicine Finland (FIMM), HiLIFE, University of Helsinki, Helsinki; Broad Institute of MIT and Harvard; Massachusetts General Hospital, Boston MA, United States	Steering Committee	Steering Committee
Bridget Riley-Gills	Abbvie, Chicago, IL, United States	Steering Committee	Pharmaceutical companies
Howard Jacob	Abbvie, Chicago, IL, United States	Steering Committee	Pharmaceutical companies
Dirk Paul	Astra Zeneca, Cambridge, United Kingdom	Steering Committee	Pharmaceutical companies
Athena Matakidou	Astra Zeneca, Cambridge, United Kingdom	Steering Committee	Pharmaceutical companies
Adam Platt	Astra Zeneca, Cambridge, United Kingdom	Steering Committee	Pharmaceutical companies
Heiko Runz	Biogen, Cambridge, MA, United States	Steering Committee	Pharmaceutical companies
Sally John Biogen,	Cambridge, MA, United States	Steering Committee	Pharmaceutical companies
George Okafo	Boehringer Ingelheim, Ingelheim am Rhein, Germany	Steering Committee	Pharmaceutical companies
Nathan Lawless	Boehringer Ingelheim, Ingelheim am Rhein, Germany	Steering Committee	Pharmaceutical companies
Heli Salminen-Mankonen	Boehringer Ingelheim, Ingelheim am Rhein, German	Steering Committee	Pharmaceutical companies
Robert Plenge	Bristol Myers Squibb, New York, NY, United States	Steering Committee	Pharmaceutical companies
Joseph Maranville	Bristol Myers Squibb, New York, NY, United States	Steering Committee	Pharmaceutical companies
Mark McCarthy	Genentech, San Francisco, CA, United States	Steering Committee	Pharmaceutical companies
Margaret G. Ehm	GlaxoSmithKline, Collegeville, PA, United States	Steering Committee	Pharmaceutical companies
Kirsi Auro	GlaxoSmithKline, Espoo, Finland	Steering Committee	Pharmaceutical companies
Simonne Longerich	Merck, Kenilworth, NJ, United States	Steering Committee	Pharmaceutical companies
Caroline Fox	Merck, Kenilworth, NJ, United States	Steering Committee	Pharmaceutical companies
Anders Mälarstig	Pfizer, New York, NY, United States	Steering Committee	Pharmaceutical companies
Katherine Klinger	Translational Sciences, Sanofi R&D, Framingham, MA, USA	Steering Committee	Pharmaceutical companies
Clement Chatelain	Translational Sciences, Sanofi R&D, Framingham, MA, USA	Steering Committee	Pharmaceutical companies
Matthias Gossel	Translational Sciences, Sanofi R&D, Framingham, MA, USA	Steering Committee	Pharmaceutical companies
Karol Estrada	Maze Therapeutics, San Francisco, CA, United States	Steering Committee	Pharmaceutical companies
Robert Graham	Maze Therapeutics, San Francisco, CA, United States	Steering Committee	Pharmaceutical companies
Robert Yang	Janssen Biotech, Beerse, Belgium	Steering Committee	Pharmaceutical companies
Chris O´Donnell	Novartis Institutes for BioMedical Research, Cambridge, MA, United States	Steering Committee	Pharmaceutical companies
Tomi P. Mäkelä	HiLIFE, University of Helsinki, Finland, Finland	Steering Committee	University of Helsinki & Biobanks
Jaakko Kaprio	Institute for Molecular Medicine Finland (FIMM), HiLIFE, University of Helsinki, Helsinki, Finland	Steering Committee	University of Helsinki & Biobanks
Petri Virolainen	FinlandAuria Biobank / University of Turku / Hospital District of Southwest Finland, Turku,	Steering Committee	University of Helsinki & Biobanks
Antti Hakanen	FinlandAuria Biobank / University of Turku / Hospital District of Southwest Finland, Turku,	Steering Committee	University of Helsinki & Biobanks
Terhi Kilpi	FinlandTHL Biobank / Finnish Institute for Health and Welfare (THL), Helsinki, Finland	Steering Committee	University of Helsinki & Biobanks
Markus Perola	THL Biobank / Finnish Institute for Health and Welfare (THL), Helsinki, Finland	Steering Committee	University of Helsinki & Biobanks
Jukka Partanen	Finnish Red Cross Blood Service / Finnish Hematology Registry and Clinical Biobank, Helsinki, Finland	Steering Committee	University of Helsinki & Biobanks
Anne Pitkäranta	Helsinki Biobank / Helsinki University and Hospital District of Helsinki and Uusimaa, Helsinki, Finland	Steering Committee	University of Helsinki & Biobanks
Taneli Raivio	Helsinki Biobank / Helsinki University and Hospital District of Helsinki and Uusimaa, Helsinki, Finland	Steering Committee	University of Helsinki & Biobanks
Raisa Serpi	Northern Finland Biobank Borealis / University of Oulu / Northern Ostrobothnia Hospital District, Oulu, Finland	Steering Committee	University of Helsinki & Biobanks
Tarja Laitinen	Finnish Clinical Biobank Tampere / University of Tampere / Pirkanmaa Hospital District, Tampere, Finland	Steering Committee	University of Helsinki & Biobanks
Veli-Matti Kosma	Biobank of Eastern Finland / University of Eastern Finland / Northern Savo Hospital District, Kuopio, Finland	Steering Committee	University of Helsinki & Biobanks
Jari Laukkanen	Central Finland Biobank / University of Jyväskylä / Central Finland Health Care District, Jyväskylä, Finland	Steering Committee	University of Helsinki & Biobanks
Marco Hautalahti	FINBB - Finnish biobank cooperative	Steering Committee	University of Helsinki & Biobanks

Outi Tuovila	Business Finland, Helsinki, Finland	Steering Committee	Other Experts/ Non-Voting Members
Raimo Pakkanen	Business Finland, Helsinki, Finland	Steering Committee	Other Experts/ Non-Voting Members
Jeffrey Waring	Abbvie, Chicago, IL, United States	Scientific Committee	Pharmaceutical companies
Bridget Riley-Gillis	Abbvie, Chicago, IL, United States	Scientific Committee	Pharmaceutical companies
Fedik Rahimov	Abbvie, Chicago, IL, United States	Scientific Committee	Pharmaceutical companies
Ioanna Tachmazidou	Astra Zeneca, Cambridge, United Kingdom	Scientific Committee	Pharmaceutical companies
Chia-Yen Chen	Biogen, Cambridge, MA, United States	Scientific Committee	Pharmaceutical companies
Heiko Runz	Biogen, Cambridge, MA, United States	Scientific Committee	Pharmaceutical companies
Zhihao Ding	Boehringer Ingelheim, Ingelheim am Rhein, Germany	Scientific Committee	Pharmaceutical companies
Marc Jung	Boehringer Ingelheim, Ingelheim am Rhein, Germany	Scientific Committee	Pharmaceutical companies
Shameek Biswas	Bristol Myers Squibb, New York, NY, United States	Scientific Committee	Pharmaceutical companies
Rion Pendergrass	Genentech, San Francisco, CA, United States	Scientific Committee	Pharmaceutical companies
Margaret G. Ehm	GlaxoSmithKline, Collegeville, PA, United States	Scientific Committee	Pharmaceutical companies
David Pulford	GlaxoSmithKline, Stevenage, United Kingdom	Scientific Committee	Pharmaceutical companies
Neha Raghavan	Merck, Kenilworth, NJ, United States	Scientific Committee	Pharmaceutical companies
Adriana Huertas-Vazquez	Merck, Kenilworth, NJ, United States	Scientific Committee	Pharmaceutical companies
Jae-Hoon Sul	Merck, Kenilworth, NJ, United States	Scientific Committee	Pharmaceutical companies
Anders Mälarstig	Pfizer, New York, NY, United States	Scientific Committee	Pharmaceutical companies
Xinli Hu	Pfizer, New York, NY, United States	Scientific Committee	Pharmaceutical companies
Åsa Hedman	Pfizer, New York, NY, United States	Scientific Committee	Pharmaceutical companies
Katherine Klinger	Translational Sciences, Sanofi R&D, Framingham, MA, USA	Scientific Committee	Pharmaceutical companies
Robert Graham	Maze Therapeutics, San Francisco, CA, United States	Scientific Committee	Pharmaceutical companies
Manuel Rivas	Maze Therapeutics, San Francisco, CA, United States	Scientific Committee	Pharmaceutical companies
Dawn Waterworth	Janssen Research & Development, LLC, Spring House, PA, United States	Scientific Committee	Pharmaceutical companies
Nicole Renaud	Novartis Institutes for BioMedical Research, Cambridge, MA, United States	Scientific Committee	Pharmaceutical companies
Ma´en Obeidat	Novartis Institutes for BioMedical Research, Cambridge, MA, United States	Scientific Committee	Pharmaceutical companies
Johanna Schleutker	Auria Biobank / Univ. of Turku / Hospital District of Southwest Finland, Turku, Finland	Scientific Committee	University of Helsinki & Biobanks
Markus Perola	THL Biobank / Finnish Institute for Health and Welfare (THL), Helsinki, Finland	Scientific Committee	University of Helsinki & Biobanks
Samuli Ripatti	Institute for Molecular Medicine Finland (FIMM), HiLIFE, University of Helsinki, Helsinki, Finland	Scientific Committee	University of Helsinki & Biobanks
Mikko Arvas	Finnish Red Cross Blood Service / Finnish Hematology Registry and Clinical Biobank, Helsinki, Finland	Scientific Committee	University of Helsinki & Biobanks
Olli Carpén	Helsinki Biobank / Helsinki University and Hospital District of Helsinki and Uusimaa, Helsinki, Finland	Scientific Committee	University of Helsinki & Biobanks
Reetta Hinttala	Northern Finland Biobank Borealis / University of Oulu / Northern Ostrobothnia Hospital District, Oulu, Finland	Scientific Committee	University of Helsinki & Biobanks
Johannes Kettunen	Northern Finland Biobank Borealis / University of Oulu / Northern Ostrobothnia Hospital District, Oulu, Finland	Scientific Committee	University of Helsinki & Biobanks
Arto Mannermaa	Biobank of Eastern Finland / University of Eastern Finland / Northern Savo Hospital District, Kuopio, Finland	Scientific Committee	University of Helsinki & Biobanks
Katriina Aalto-Setälä	Faculty of Medicine and Health Technology, Tampere University, Tampere, Finland	Scientific Committee	University of Helsinki & Biobanks
Mika Kähönen	Finnish Clinical Biobank Tampere / University of Tampere / Pirkanmaa Hospital District, Tampere, Finland	Scientific Committee	University of Helsinki & Biobanks
Jari Laukkanen	Central Finland Biobank / University of Jyväskylä / Central Finland Health Care District, Jyväskylä, Finland	Scientific Committee	University of Helsinki & Biobanks
Johanna Mäkelä	FINBB - Finnish biobank cooperative	Scientific Committee	University of Helsinki & Biobanks
Reetta Kälviäinen	Northern Savo Hospital District, Kuopio, Finland	Clinical Groups	Neurology Group
Valtteri Julkunen	Northern Savo Hospital District, Kuopio, Finland	Clinical Groups	Neurology Group
Hilkka Soininen	Northern Savo Hospital District, Kuopio, Finland	Clinical Groups	Neurology Group
Anne Remes	Northern Ostrobothnia Hospital District, Oulu, Finland	Clinical Groups	Neurology Group
Mikko Hiltunen	University of Eastern Finland, Kuopio, Finland	Clinical Groups	Neurology Group
Jukka Peltola	Pirkanmaa Hospital District, Tampere, Finland	Clinical Groups	Neurology Group

Minna Raivio	Hospital District of Helsinki and Uusimaa, Helsinki, Finland	Clinical Groups	Neurology Group
Pentti Tienari	Hospital District of Helsinki and Uusimaa, Helsinki, Finland	Clinical Groups	Neurology Group
Juha Rinne	Hospital District of Southwest Finland, Turku, Finland	Clinical Groups	Neurology Group
Roosa Kallionpää	Hospital District of Southwest Finland, Turku, Finland	Clinical Groups	Neurology Group
Juulia Partanen	Institute for Molecular Medicine Finland, HiLIFE, University of Helsinki, Finland	Clinical Groups	Neurology Group
Ali Abbasi	Abbvie, Chicago, IL, United States	Clinical Groups	Neurology Group
Adam Ziemann	Abbvie, Chicago, IL, United States	Clinical Groups	Neurology Group
Nizar Smaoui	Abbvie, Chicago, IL, United States	Clinical Groups	Neurology Group
Anne Lehtonen	Abbvie, Chicago, IL, United States	Clinical Groups	Neurology Group
Susan Eaton	Biogen, Cambridge, MA, United States	Clinical Groups	Neurology Group
Heiko Runz	Biogen, Cambridge, MA, United States	Clinical Groups	Neurology Group
Sanni Lahdenperä	Biogen, Cambridge, MA, United States	Clinical Groups	Neurology Group
Shameek Biswas	Bristol Myers Squibb, New York, NY, United States	Clinical Groups	Neurology Group
Natalie Bowers	Genentech, San Francisco, CA, United States	Clinical Groups	Neurology Group
Edmond Teng	Genentech, San Francisco, CA, United States	Clinical Groups	Neurology Group
Rion Pendergrass	Genentech, San Francisco, CA, United States	Clinical Groups	Neurology Group
Fanli Xu	GlaxoSmithKline, Brentford, United Kingdom	Clinical Groups	Neurology Group
David Pulford	GlaxoSmithKline, Stevenage, United Kingdom	Clinical Groups	Neurology Group
Kirsi Auro	GlaxoSmithKline, Espoo, Finland	Clinical Groups	Neurology Group
Laura Addis	GlaxoSmithKline, Brentford, United Kingdom	Clinical Groups	Neurology Group
John Eicher	GlaxoSmithKline, Brentford, United Kingdom	Clinical Groups	Neurology Group
Qingqin S Li	Janssen Research & Development, LLC, Titusville, NJ 08560, United States	Clinical Groups	Neurology Group
Karen He	Janssen Research & Development, LLC, Spring House, PA, United States	Clinical Groups	Neurology Group
Ekaterina Khramtsova	Janssen Research & Development, LLC, Spring House, PA, United States	Clinical Groups	Neurology Group
Neha Raghavan	Merck, Kenilworth, NJ, United States	Clinical Groups	Neurology Group
Martti Färkkilä	Hospital District of Helsinki and Uusimaa, Helsinki, Finland	Clinical Groups	Gastroenterology Group
Jukka Koskela	Hospital District of Helsinki and Uusimaa, Helsinki, Finland	Clinical Groups	Gastroenterology Group
Sampsa Pikkarainen	Hospital District of Helsinki and Uusimaa, Helsinki, Finland	Clinical Groups	Gastroenterology Group
Airi Jussila	Pirkanmaa Hospital District, Tampere, Finland	Clinical Groups	Gastroenterology Group
Katri Kaukinen	Pirkanmaa Hospital District, Tampere, Finland	Clinical Groups	Gastroenterology Group
Timo Blomster	Northern Ostrobothnia Hospital District, Oulu, Finland	Clinical Groups	Gastroenterology Group
Mikko Kiviniemi	Northern Savo Hospital District, Kuopio, Finland	Clinical Groups	Gastroenterology Group
Markku Voutilainen	Hospital District of Southwest Finland, Turku, Finland	Clinical Groups	Gastroenterology Group
Mark Daly	Institute for Molecular Medicine Finland (FIMM), HiLIFE, University of Helsinki, Helsinki; Broad Institute of MIT and Harvard; Massachusetts General Hospital, Boston MA, United States	Clinical Groups	Gastroenterology Group
Ali Abbasi	Abbvie, Chicago, IL, United States	Clinical Groups	Gastroenterology Group
Jeffrey Waring	Abbvie, Chicago, IL, United States	Clinical Groups	Gastroenterology Group
Nizar Smaoui	Abbvie, Chicago, IL, United States	Clinical Groups	Gastroenterology Group
Fedik Rahimov	Abbvie, Chicago, IL, United States	Clinical Groups	Gastroenterology Group
Anne Lehtonen	Abbvie, Chicago, IL, United States	Clinical Groups	Gastroenterology Group
Tim Lu	Genentech, San Francisco, CA, United States	Clinical Groups	Gastroenterology Group
Natalie Bowers	Genentech, San Francisco, CA, United States	Clinical Groups	Gastroenterology Group
Rion Pendergrass	Genentech, San Francisco, CA, United States	Clinical Groups	Gastroenterology Group
Linda McCarthy	GlaxoSmithKline, Brentford, United Kingdom	Clinical Groups	Gastroenterology Group
Amy Hart	Janssen Research & Development, LLC, Spring House, PA, United States	Clinical Groups	Gastroenterology Group

Meijian Guan	Janssen Research & Development, LLC, Spring House, PA, United States	Clinical Groups	Gastroenterology Group
Jason Miller	Merck, Kenilworth, NJ, United States	Clinical Groups	Gastroenterology Group
Kirsi Kalpala	Pfizer, New York, NY, United States	Clinical Groups	Gastroenterology Group
Melissa Miller	Pfizer, New York, NY, United States	Clinical Groups	Gastroenterology Group
Xinli Hu	Pfizer, New York, NY, United States	Clinical Groups	Gastroenterology Group
Kari Eklund	Hospital District of Helsinki and Uusimaa, Helsinki, Finland	Clinical Groups	Rheumatology Group
Antti Palomäki	Hospital District of Southwest Finland, Turku, Finland	Clinical Groups	Rheumatology Group
Pia Isomäki	Pirkanmaa Hospital District, Tampere, Finland	Clinical Groups	Rheumatology Group
Laura Pirilä	Hospital District of Southwest Finland, Turku, Finland	Clinical Groups	Rheumatology Group
Oili Kaipiainen-Seppänen	Northern Savo Hospital District, Kuopio, Finland	Clinical Groups	Rheumatology Group
Johanna Huhtakangas	Northern Ostrobothnia Hospital District, Oulu, Finland	Clinical Groups	Rheumatology Group
Nina Mars	Institute for Molecular Medicine Finland (FIMM), HiLIFE, University of Helsinki, Helsinki, Finland	Clinical Groups	Rheumatology Group
Ali Abbasi	Abbvie, Chicago, IL, United States	Clinical Groups	Rheumatology Group
Jeffrey Waring	Abbvie, Chicago, IL, United States	Clinical Groups	Rheumatology Group
Fedik Rahimov	Abbvie, Chicago, IL, United States	Clinical Groups	Rheumatology Group
Apinya Lertratanakul	Abbvie, Chicago, IL, United States	Clinical Groups	Rheumatology Group
Nizar Smaoui	Abbvie, Chicago, IL, United States	Clinical Groups	Rheumatology Group
Anne Lehtonen	Abbvie, Chicago, IL, United States	Clinical Groups	Rheumatology Group
Marla Hochfeld	Bristol Myers Squibb, New York, NY, United States	Clinical Groups	Rheumatology Group
Natalie Bowers	Genentech, San Francisco, CA, United States	Clinical Groups	Rheumatology Group
Rion Pendergrass	Genentech, San Francisco, CA, United States	Clinical Groups	Rheumatology Group
Jorge Esparza Gordillo	GlaxoSmithKline, Brentford, United Kingdom	Clinical Groups	Rheumatology Group
Kirsi Auro	GlaxoSmithKline, Espoo, Finland	Clinical Groups	Rheumatology Group
Dawn Waterworth	Janssen Research & Development, LLC, Spring House, PA, United States	Clinical Groups	Rheumatology Group
Fabiana Farias	Merck, Kenilworth, NJ, United States	Clinical Groups	Rheumatology Group
Kirsi Kalpala	Pfizer, New York, NY, United States	Clinical Groups	Rheumatology Group
Nan Bing	Pfizer, New York, NY, United States	Clinical Groups	Rheumatology Group
Xinli Hu	Pfizer, New York, NY, United States	Clinical Groups	Rheumatology Group
Tarja Laitinen	Pirkanmaa Hospital District, Tampere, Finland	Clinical Groups	Pulmonology Group
Margit Pelkonen	Northern Savo Hospital District, Kuopio, Finland	Clinical Groups	Pulmonology Group
Paula Kauppi	Hospital District of Helsinki and Uusimaa, Helsinki, Finland	Clinical Groups	Pulmonology Group
Hannu Kankaanranta	University of Gothenburg, Gothenburg, Sweden/ Seinäjoki Central Hospital, Seinäjoki, Finland/ Tampere University, Tampere, Finland	Clinical Groups	Pulmonology Group
Terttu Harju	Northern Ostrobothnia Hospital District, Oulu, Finland	Clinical Groups	Pulmonology Group
Riitta Lahesmaa	Hospital District of Southwest Finland, Turku, Finland	Clinical Groups	Pulmonology Group
Nizar Smaoui	Abbvie, Chicago, IL, United States	Clinical Groups	Pulmonology Group
Glenda Lassi	Astra Zeneca, Cambridge, United Kingdom	Clinical Groups	Pulmonology Group
Susan Eaton	Biogen, Cambridge, MA, United States	Clinical Groups	Pulmonology Group
Hubert Chen	Genentech, San Francisco, CA, United States	Clinical Groups	Pulmonology Group
Rion Pendergrass	Genentech, San Francisco, CA, United States	Clinical Groups	Pulmonology Group
Natalie Bowers	Genentech, San Francisco, CA, United States	Clinical Groups	Pulmonology Group
Joanna Betts	GlaxoSmithKline, Brentford, United Kingdom	Clinical Groups	Pulmonology Group
Kirsi Auro	GlaxoSmithKline, Espoo, Finland	Clinical Groups	Pulmonology Group
Rajashree Mishra	GlaxoSmithKline, Brentford, United Kingdom	Clinical Groups	Pulmonology Group
Majd Mouded	Novartis, Basel, Switzerland	Clinical Groups	Pulmonology Group

Debby Ngo	Novartis, Basel, Switzerland	Clinical Groups	Pulmonology Group
Teemu Niiranen	Finnish Institute for Health and Welfare (THL), Helsinki, Finland	Clinical Groups	Cardiometabolic Diseases Group
Felix Vaura	Finnish Institute for Health and Welfare (THL), Helsinki, Finland	Clinical Groups	Cardiometabolic Diseases Group
Veikko Salomaa	Finnish Institute for Health and Welfare (THL), Helsinki, Finland	Clinical Groups	Cardiometabolic Diseases Group
Kaj Metsärinne	Hospital District of Southwest Finland, Turku, Finland	Clinical Groups	Cardiometabolic Diseases Group
Jenni Aittokallio	Hospital District of Southwest Finland, Turku, Finland	Clinical Groups	Cardiometabolic Diseases Group
Jussi Hernesniemi	Pirkanmaa Hospital District, Tampere, Finland	Clinical Groups	Cardiometabolic Diseases Group
Daniel Gordin	Hospital District of Helsinki and Uusimaa, Helsinki, Finland	Clinical Groups	Cardiometabolic Diseases Group
Juha Sinisalo	Hospital District of Helsinki and Uusimaa, Helsinki, Finland	Clinical Groups	Cardiometabolic Diseases Group
Marja-Riitta Taskinen	Hospital District of Helsinki and Uusimaa, Helsinki, Finland	Clinical Groups	Cardiometabolic Diseases Group
Tiinamaija Tuomi	Hospital District of Helsinki and Uusimaa, Helsinki, Finland	Clinical Groups	Cardiometabolic Diseases Group
Timo Hiltunen	Hospital District of Helsinki and Uusimaa, Helsinki, Finland	Clinical Groups	Cardiometabolic Diseases Group
Jari Laukkanen	Central Finland Health Care District, Jyväskylä, Finland	Clinical Groups	Cardiometabolic Diseases Group
Amanda Elliott	Institute for Molecular Medicine Finland (FIMM), HiLIFE, University of Helsinki, Helsinki, Finland; Broad Institute, Cambridge, MA, USA and Massachusetts General Hospital, Boston, MA, USA	Clinical Groups	Cardiometabolic Diseases Group
Mary Pat Reeve	Institute for Molecular Medicine Finland (FIMM), HiLIFE, University of Helsinki, Helsinki, Finland	Clinical Groups	Cardiometabolic Diseases Group
Sanni Ruotsalainen	Institute for Molecular Medicine Finland (FIMM), HiLIFE, University of Helsinki, Helsinki, Finland	Clinical Groups	Cardiometabolic Diseases Group
Benjamin Challis	Astra Zeneca, Cambridge, United Kingdom	Clinical Groups	Cardiometabolic Diseases Group
Dirk Paul	Astra Zeneca, Cambridge, United Kingdom	Clinical Groups	Cardiometabolic Diseases Group
Natalie Bowers	Genentech, San Francisco, CA, United States	Clinical Groups	Cardiometabolic Diseases Group
Rion Pendergrass	Genentech, San Francisco, CA, United States	Clinical Groups	Cardiometabolic Diseases Group
Audrey Chu	GlaxoSmithKline, Brentford, United Kingdom	Clinical Groups	Cardiometabolic Diseases Group
Kirsi Auro	GlaxoSmithKline, Espoo, Finland	Clinical Groups	Cardiometabolic Diseases Group
Dermot Reilly	Janssen Research & Development, LLC, Boston, MA, United States	Clinical Groups	Cardiometabolic Diseases Group
Mike Mendelson	Novartis, Boston, MA, United States	Clinical Groups	Cardiometabolic Diseases Group
Jaakko Parkkinen	Pfizer, New York, NY, United States	Clinical Groups	Cardiometabolic Diseases Group
Melissa Miller	Pfizer, New York, NY, United States	Clinical Groups	Cardiometabolic Diseases Group
Tuomo Meretoja	Hospital District of Helsinki and Uusimaa, Helsinki, Finland	Clinical Groups	Oncology Group
Heikki Joensuu	Hospital District of Helsinki and Uusimaa, Helsinki, Finland	Clinical Groups	Oncology Group
Olli Carpén	Hospital District of Helsinki and Uusimaa, Helsinki, Finland	Clinical Groups	Oncology Group
Johanna Mattson	Hospital District of Helsinki and Uusimaa, Helsinki, Finland	Clinical Groups	Oncology Group
Eveliina Salminen	Hospital District of Helsinki and Uusimaa, Helsinki, Finland	Clinical Groups	Oncology Group
Annika Auranen	Pirkanmaa Hospital District , Tampere, Finland	Clinical Groups	Oncology Group
Peeter Karihtala	Northern Ostrobothnia Hospital District, Oulu, Finland	Clinical Groups	Oncology Group
Päivi Auvinen	Northern Savo Hospital District, Kuopio, Finland	Clinical Groups	Oncology Group
Klaus Elenius	Hospital District of Southwest Finland, Turku, Finland	Clinical Groups	Oncology Group
Johanna Schleutker	Hospital District of Southwest Finland, Turku, Finland	Clinical Groups	Oncology Group
Esa Pitkänen	Institute for Molecular Medicine Finland (FIMM), HiLIFE, University of Helsinki, Helsinki, Finland	Clinical Groups	Oncology Group
Mark Daly	Institute for Molecular Medicine Finland (FIMM), HiLIFE, University of Helsinki, Helsinki; Broad Institute of MIT and Harvard; Massachusetts General Hospital, Boston MA, United States	Clinical Groups	Oncology Group
Nina Mars	Institute for Molecular Medicine Finland (FIMM), HiLIFE, University of Helsinki, Helsinki, Finland	Clinical Groups	Oncology Group
Relja Popovic	Abbvie, Chicago, IL, United States	Clinical Groups	Oncology Group
Jeffrey Waring	Abbvie, Chicago, IL, United States	Clinical Groups	Oncology Group
Bridget Riley-Gillis	Abbvie, Chicago, IL, United States	Clinical Groups	Oncology Group
Anne Lehtonen	Abbvie, Chicago, IL, United States	Clinical Groups	Oncology Group
Jennifer Schutzman	Genentech, San Francisco, CA, United States	Clinical Groups	Oncology Group

Natalie Bowers	Genentech, San Francisco, CA, United States	Clinical Groups	Oncology Group
Rion Pendergrass	Genentech, San Francisco, CA, United States	Clinical Groups	Oncology Group
Diptee Kulkarni	GlaxoSmithKline, Brentford, United Kingdom	Clinical Groups	Oncology Group
Kirsi Auro	GlaxoSmithKline, Espoo, Finland	Clinical Groups	Oncology Group
Alessandro Porello	Janssen Research & Development, LLC, Spring House, PA, United States	Clinical Groups	Oncology Group
Andrey Loboda	Merck, Kenilworth, NJ, United States	Clinical Groups	Oncology Group
Heli Lehtonen	Pfizer, New York, NY, United States	Clinical Groups	Oncology Group
Stefan McDonough	Pfizer, New York, NY, United States	Clinical Groups	Oncology Group
Sauli Vuoti	Janssen-Cilag Oy, Espoo, Finland	Clinical Groups	Oncology Group
Kai Kaarniranta	Northern Savo Hospital District, Kuopio, Finland	Clinical Groups	Opthalmology Group
Joni A Turunen	Helsinki University Hospital and University of Helsinki, Helsinki, Finland; Eye Genetics Group, Folkhälsan Research Center, Helsinki, Finland	Clinical Groups	Opthalmology Group
Terhi Ollila	Hospital District of Helsinki and Uusimaa, Helsinki, Finland	Clinical Groups	Opthalmology Group
Hannu Uusitalo	Pirkanmaa Hospital District, Tampere, Finland	Clinical Groups	Opthalmology Group
Esa Pitkänen	Institute for Molecular Medicine Finland (FIMM), HiLIFE, University of Helsinki, Helsinki, Finland	Clinical Groups	Opthalmology Group
Juha Karjalainen	Institute for Molecular Medicine Finland (FIMM), HiLIFE, University of Helsinki, Helsinki, Finland	Clinical Groups	Opthalmology Group
Mengzhen Liu	Abbvie, Chicago, IL, United States	Clinical Groups	Opthalmology Group
Heiko Runz	Biogen, Cambridge, MA, United States	Clinical Groups	Opthalmology Group
Stephanie Loomis s	Biogen, Cambridge, MA, United State	Clinical Groups	Opthalmology Group
Erich Strauss	Genentech, San Francisco, CA, United States	Clinical Groups	Opthalmology Group
Natalie Bowers	Genentech, San Francisco, CA, United States	Clinical Groups	Opthalmology Group
Hao Chen	Genentech, San Francisco, CA, United States	Clinical Groups	Opthalmology Group
Rion Pendergrass	Genentech, San Francisco, CA, United States	Clinical Groups	Opthalmology Group
Kaisa Tasanen	Northern Ostrobothnia Hospital District, Oulu, Finland	Clinical Groups	Dermatology Group
Laura Huilaja	Northern Ostrobothnia Hospital District, Oulu, Finland	Clinical Groups	Dermatology Group
Katariina Hannula-Jouppi	Hospital District of Helsinki and Uusimaa, Helsinki, Finland	Clinical Groups	Dermatology Group
Teea Salmi	Pirkanmaa Hospital District, Tampere, Finland	Clinical Groups	Dermatology Group
Sirkku Peltonen	Hospital District of Southwest Finland, Turku, Finland	Clinical Groups	Dermatology Group
Leena Koulu	Hospital District of Southwest Finland, Turku, Finland	Clinical Groups	Dermatology Group
Nizar Smaoui	Abbvie, Chicago, IL, United States	Clinical Groups	Dermatology Group
Fedik Rahimov	Abbvie, Chicago, IL, United States	Clinical Groups	Dermatology Group
Anne Lehtonen	Abbvie, Chicago, IL, United States	Clinical Groups	Dermatology Group
David Choy	Genentech, San Francisco, CA, United States	Clinical Groups	Dermatology Group
Rion Pendergrass	Genentech, San Francisco, CA, United States	Clinical Groups	Dermatology Group
Dawn Waterworth	Janssen Research & Development, LLC, Spring House, PA, United States	Clinical Groups	Dermatology Group
Kirsi Kalpala	Pfizer, New York, NY, United States	Clinical Groups	Dermatology Group
Ying Wu	Pfizer, New York, NY, United States	Clinical Groups	Dermatology Group
Pirkko Pussinen	Hospital District of Helsinki and Uusimaa, Helsinki, Finland	Clinical Groups	Odontology Group
Aino Salminen	Hospital District of Helsinki and Uusimaa, Helsinki, Finland	Clinical Groups	Odontology Group
Tuula Salo	Hospital District of Helsinki and Uusimaa, Helsinki, Finland	Clinical Groups	Odontology Group
David Rice	Hospital District of Helsinki and Uusimaa, Helsinki, Finland	Clinical Groups	Odontology Group
Pekka Nieminen	Hospital District of Helsinki and Uusimaa, Helsinki, Finland	Clinical Groups	Odontology Group
Ulla Palotie	Hospital District of Helsinki and Uusimaa, Helsinki, Finland	Clinical Groups	Odontology Group
Maria Siponen	Northern Savo Hospital District, Kuopio, Finland	Clinical Groups	Odontology Group
Liisa Suominen	Northern Savo Hospital District, Kuopio, Finland	Clinical Groups	Odontology Group

Päivi Mäntylä	Northern Savo Hospital District, Kuopio, Finland	Clinical Groups	Odontology Group
Ulvi Gursoy	Hospital District of Southwest Finland, Turku, Finland	Clinical Groups	Odontology Group
Vuokko Anttonen	Northern Ostrobothnia Hospital District, Oulu, Finland	Clinical Groups	Odontology Group
Kirsi Sipilä	Research Unit of Oral Health Sciences Faculty of Medicine, University of Oulu, Oulu, Finland; Medical Research Center, Oulu, Oulu University Hospital and University of Oulu, Oulu, Finland	Clinical Groups	Odontology Group
Rion Pendergrass	Genentech, San Francisco, CA, United States	Clinical Groups	Odontology Group
Hannele Laivuori	Institute for Molecular Medicine Finland (FIMM), HiLIFE, University of Helsinki, Helsinki, Finland	Clinical Groups	Women's Health and Reproduction Group
Venla Kurra	Pirkanmaa Hospital District, Tampere, Finland	Clinical Groups	Women's Health and Reproduction Group
Laura Kotaniemi-Talonen	Pirkanmaa Hospital District, Tampere, Finland	Clinical Groups	Women's Health and Reproduction Group
Oskari Heikinheimo	Hospital District of Helsinki and Uusimaa, Helsinki, Finland	Clinical Groups	Women's Health and Reproduction Group
Ilkka Kalliala	Hospital District of Helsinki and Uusimaa, Helsinki, Finland	Clinical Groups	Women's Health and Reproduction Group
Lauri Aaltonen	Hospital District of Helsinki and Uusimaa, Helsinki, Finland	Clinical Groups	Women's Health and Reproduction Group
Varpu Jokimaa	Hospital District of Southwest Finland, Turku, Finland	Clinical Groups	Women's Health and Reproduction Group
Terhi Piltonen	Northern Ostrobothnia Hospital District, Oulu, Finland	Clinical Groups	Women's Health and Reproduction Group
Johannes Kettunen	Northern Ostrobothnia Hospital District, Oulu, Finland	Clinical Groups	Women's Health and Reproduction Group
Marja Vääräsmäki	Northern Ostrobothnia Hospital District, Oulu, Finland	Clinical Groups	Women's Health and Reproduction Group
Outi Uimari	Northern Ostrobothnia Hospital District, Oulu, Finland	Clinical Groups	Women's Health and Reproduction Group
Laure Morin-Papunen	Northern Ostrobothnia Hospital District, Oulu, Finland	Clinical Groups	Women's Health and Reproduction Group
Maarit Niinimäki	Northern Ostrobothnia Hospital District, Oulu, Finland	Clinical Groups	Women's Health and Reproduction Group
Katja Kivinen	Institute for Molecular Medicine Finland (FIMM), HiLIFE, University of Helsinki, Helsinki, Finland	Clinical Groups	Women's Health and Reproduction Group
Elisabeth Widen	Institute for Molecular Medicine Finland (FIMM), HiLIFE, University of Helsinki, Helsinki, Finland	Clinical Groups	Women's Health and Reproduction Group
Mary Pat Reeve	Institute for Molecular Medicine Finland (FIMM), HiLIFE, University of Helsinki, Helsinki, Finland	Clinical Groups	Women's Health and Reproduction Group
Mark Daly	Institute for Molecular Medicine Finland (FIMM), HiLIFE, University of Helsinki, Helsinki; Broad Institute of MIT and Harvard; Massachusetts General Hospital, Boston MA, United States	Clinical Groups	Women's Health and Reproduction Group
Taru Tukiainen	Institute for Molecular Medicine Finland (FIMM), HiLIFE, University of Helsinki, Helsinki, Finland	Clinical Groups	Women's Health and Reproduction Group
Niko Välimäki	University of Helsinki, Helsinki, Finland	Clinical Groups	Women's Health and Reproduction Group
Eija Laakkonen	University of Jyväskylä, Jyväskylä, Finland	Clinical Groups	Women's Health and Reproduction Group
Jaakko Tyrmi	University of Oulu, Oulu, Finland / University of Tampere, Tampere, Finland	Clinical Groups	Women's Health and Reproduction Group
Heidi Silven	University of Oulu, Oulu, Finland	Clinical Groups	Women's Health and Reproduction Group
Eeva Sliz	University of Oulu, Oulu, Finland	Clinical Groups	Women's Health and Reproduction Group
Riikka Arffman	University of Oulu, Oulu, Finland	Clinical Groups	Women's Health and Reproduction Group
Susanna Savukoski	University of Oulu, Oulu, Finland	Clinical Groups	Women's Health and Reproduction Group
Triin Laisk	Estonian biobank, Tartu, Estonia	Clinical Groups	Women's Health and Reproduction Group
Natalia Pujol	Estonian biobank, Tartu, Estonia	Clinical Groups	Women's Health and Reproduction Group
Mengzhen Liu	Abbvie, Chicago, IL, United States	Clinical Groups	Women's Health and Reproduction Group
Bridget Riley-Gillis	Abbvie, Chicago, IL, United States	Clinical Groups	Women's Health and Reproduction Group
Rion Pendergrass	Genentech, San Francisco, CA, United States	Clinical Groups	Women's Health and Reproduction Group
Janet Kumar	GlaxoSmithKline, Collegeville, PA, United States	Clinical Groups	Women's Health and Reproduction Group
Kirsi Auro	GlaxoSmithKline, Espoo, Finland	Clinical Groups	Women's Health and Reproduction Group
Iiris Hovatta	University of Helsinki, Finland	Clinical Groups	Depression group
Chia-Yen Chen	Biogen, Cambridge, MA, United States	Clinical Groups	Depression group
Erkki Isometsä	Hospital District of Helsinki and Uusimaa, Helsinki, Finland	Clinical Groups	Depression group
Hanna Ollila	Institute for Molecular Medicine Finland (FIMM), HiLIFE, University of Helsinki, Helsinki, Finland	Clinical Groups	Depression group
Jaana Suvisaari	Finnish Institute for Health and Welfare (THL), Helsinki, Finland	Clinical Groups	Depression group
Thomas Damm Als	Aarhus University, Denmark	Clinical Groups	Depression group
Antti Mäkitie	Department of Otorhinolaryngology - Head and Neck Surgery, University of Helsinki and Helsinki University Hospital, Helsinki, Finland	Clinical Groups	ENT (ear, nose and throath) Group

Argyro Bizaki-Vallaskanga	Pirkanmaa Hospital District, Tampere, Finland	Clinical Groups	ENT (ear, nose and throath) Group
Sanna Toppila-Salmi	University of Helsinki, Finland	Clinical Groups	ENT (ear, nose and throath) Group
Elmo Saarentaus	Institute for Molecular Medicine Finland (FIMM), HiLIFE, University of Helsinki, Helsinki, Finland	Clinical Groups	ENT (ear, nose and throath) Group
Tytti Willberg	Hospital District of Southwest Finland, Turku, Finland	Clinical Groups	ENT (ear, nose and throath) Group
Antti Aarnisalo	Hospital District of Helsinki and Uusimaa, Helsinki, Finland	Clinical Groups	ENT (ear, nose and throath) Group
Eveliina Salminen	Hospital District of Helsinki and Uusimaa, Helsinki, Finland	Clinical Groups	ENT (ear, nose and throath) Group
Elisa Rahikkala	Northern Ostrobothnia Hospital District, Oulu, Finland	Clinical Groups	ENT (ear, nose and throath) Group
Johannes Kettunen	Northern Ostrobothnia Hospital District, Oulu, Finland	Clinical Groups	ENT (ear, nose and throath) Group
Kristiina Aittomäki	Department of Medical Genetics, Helsinki University Central Hospital, Helsinki, Finland	Clinical Groups	POI (premature ovarian failure) Group
Fredrik Åberg	Transplantation and Liver Surgery Clinic, Helsinki University Hospital, Helsinki University, Helsinki, Finland	Clinical Groups	LiverScore Group
Aarno Palotie	Institute for Molecular Medicine, Finland (FIMM), HiLIFE, University of Helsinki, Helsinki, Finland; Broad Institute of MIT and Harvard; Massachusetts General Hospital	FinnGen Analysis working group	FinnGen Analysis working group
Mark Daly	Institute for Molecular Medicine Finland (FIMM), HiLIFE, University of Helsinki, Helsinki; Broad Institute of MIT and Harvard; Massachusetts General Hospital, Boston MA, United States	FinnGen Analysis working group	FinnGen Analysis working group
Samuli Ripatti	Institute for Molecular Medicine Finland (FIMM), HiLIFE, University of Helsinki, Helsinki, Finland	FinnGen Analysis working group	FinnGen Analysis working group
Aki Havulinna	Institute for Molecular Medicine Finland (FIMM), HiLIFE, University of Helsinki, Helsinki, Finland	FinnGen Analysis working group	FinnGen Analysis working group
Mitja Kurki	Institute for Molecular Medicine Finland (FIMM), HiLIFE, University of Helsinki, Helsinki, Finland	FinnGen Analysis working group	FinnGen Analysis working group
Juha Mehtonen	Institute for Molecular Medicine Finland (FIMM), HiLIFE, University of Helsinki, Helsinki, Finland	FinnGen Analysis working group	FinnGen Analysis working group
Priit Palta	Institute for Molecular Medicine Finland (FIMM), HiLIFE, University of Helsinki, Helsinki, Finland	FinnGen Analysis working group	FinnGen Analysis working group
Juha Karjalainen	Institute for Molecular Medicine Finland (FIMM), HiLIFE, University of Helsinki, Helsinki, Finland	FinnGen Analysis working group	FinnGen Analysis working group
Pietro Della Briotta Parolo	Institute for Molecular Medicine Finland (FIMM), HiLIFE, University of Helsinki, Helsinki, Finland	FinnGen Analysis working group	FinnGen Analysis working group
Wei Zhou	Broad Institute, Cambridge, MA, United States	FinnGen Analysis working group	FinnGen Analysis working group
Mutaamba Maasha	Broad Institute, Cambridge, MA, United States	FinnGen Analysis working group	FinnGen Analysis working group
Susanna Lemmelä	Institute for Molecular Medicine Finland (FIMM), HiLIFE, University of Helsinki, Helsinki, Finland	FinnGen Analysis working group	FinnGen Analysis working group
Manuel Rivas	University of Stanford, Stanford, CA, United States	FinnGen Analysis working group	FinnGen Analysis working group
Aoxing Liu	Institute for Molecular Medicine Finland (FIMM), HiLIFE, University of Helsinki, Helsinki, Finland	FinnGen Analysis working group	FinnGen Analysis working group
Arto Lehisto	Institute for Molecular Medicine Finland (FIMM), HiLIFE, University of Helsinki, Helsinki, Finland	FinnGen Analysis working group	FinnGen Analysis working group
Andrea Ganna	Institute for Molecular Medicine Finland (FIMM), HiLIFE, University of Helsinki, Helsinki, Finland	FinnGen Analysis working group	FinnGen Analysis working group
Vincent Llorens	Institute for Molecular Medicine Finland (FIMM), HiLIFE, University of Helsinki, Helsinki, Finland	FinnGen Analysis working group	FinnGen Analysis working group
Hannele Laivuori	Institute for Molecular Medicine Finland (FIMM), HiLIFE, University of Helsinki, Helsinki, Finland	FinnGen Analysis working group	FinnGen Analysis working group
Mary Pat Reeve	Institute for Molecular Medicine Finland (FIMM), HiLIFE, University of Helsinki, Helsinki, Finland	FinnGen Analysis working group	FinnGen Analysis working group
Henrike Heyne	Institute for Molecular Medicine Finland (FIMM), HiLIFE, University of Helsinki, Helsinki, Finland	FinnGen Analysis working group	FinnGen Analysis working group
Nina Mars	Institute for Molecular Medicine Finland (FIMM), HiLIFE, University of Helsinki, Helsinki, Finland	FinnGen Analysis working group	FinnGen Analysis working group
Joel Rämö	Institute for Molecular Medicine Finland (FIMM), HiLIFE, University of Helsinki, Helsinki, Finland	FinnGen Analysis working group	FinnGen Analysis working group
Hanna Ollila	Institute for Molecular Medicine Finland (FIMM), HiLIFE, University of Helsinki, Helsinki, Finland	FinnGen Analysis working group	FinnGen Analysis working group
Elmo Saarentaus	Institute for Molecular Medicine Finland (FIMM), HiLIFE, University of Helsinki, Helsinki, Finland	FinnGen Analysis working group	FinnGen Analysis working group
Rodos Rodosthenous	Institute for Molecular Medicine Finland (FIMM), HiLIFE, University of Helsinki, Helsinki, Finland	FinnGen Analysis working group	FinnGen Analysis working group
Shabbeer Hassan	Institute for Molecular Medicine Finland (FIMM), HiLIFE, University of Helsinki, Helsinki, Finland	FinnGen Analysis working group	FinnGen Analysis working group
Satu Strausz	Institute for Molecular Medicine Finland (FIMM), HiLIFE, University of Helsinki, Helsinki, Finland	FinnGen Analysis working group	FinnGen Analysis working group
Taru Tukiainen	Institute for Molecular Medicine Finland (FIMM), HiLIFE, University of Helsinki, Helsinki, Finland	FinnGen Analysis working group	FinnGen Analysis working group
Tuula Palotie	University of Helsinki and Hospital District of Helsinki and Uusimaa, Helsinki, Finland	FinnGen Analysis working group	FinnGen Analysis working group
Kimmo Palin	University of Helsinki, Helsinki, Finland	FinnGen Analysis working group	FinnGen Analysis working group
Javier Garcia-Tabuenca	University of Tampere, Tampere, Finland	FinnGen Analysis working group	FinnGen Analysis working group
Harri Siirtola	University of Tampere, Tampere, Finland	FinnGen Analysis working group	FinnGen Analysis working group
Tuomo Kiiskinen	Institute for Molecular Medicine Finland (FIMM), HiLIFE, University of Helsinki, Helsinki, Finland	FinnGen Analysis working group	FinnGen Analysis working group
Jiwoo Lee	Institute for Molecular Medicine Finland (FIMM), HiLIFE, University of Helsinki, Helsinki, Finland; Broad Institute, Cambridge, MA, United States	FinnGen Analysis working group	FinnGen Analysis working group

Kristin Tsuo	Institute for Molecular Medicine Finland (FIMM), HiLIFE, University of Helsinki, Helsinki, Finland; Broad Institute, Cambridge, MA, United States	FinnGen Analysis working group	FinnGen Analysis working group
Amanda Elliott	Institute for Molecular Medicine Finland (FIMM), HiLIFE, University of Helsinki, Helsinki, Finland; Broad Institute, Cambridge, MA, USA and Massachusetts General Hospital, Boston, MA, USA	FinnGen Analysis working group	FinnGen Analysis working group
Kati Kristiansson	THL Biobank / Finnish Institute for Health and Welfare (THL), Helsinki, Finland	FinnGen Analysis working group	FinnGen Analysis working group
Mikko Arvas	Finnish Red Cross Blood Service / Finnish Hematology Registry and Clinical Biobank, Helsinki, Finland	FinnGen Analysis working group	FinnGen Analysis working group
Kati Hyvärinen	Finnish Red Cross Blood Service, Helsinki, Finland	FinnGen Analysis working group	FinnGen Analysis working group
Jarmo Ritari	Finnish Red Cross Blood Service, Helsinki, Finland	FinnGen Analysis working group	FinnGen Analysis working group
Olli Carpén	Helsinki Biobank / Helsinki University and Hospital District of Helsinki and Uusimaa, Helsinki	FinnGen Analysis working group	FinnGen Analysis working group
Johannes Kettunen	Northern Finland Biobank Borealis / University of Oulu / Northern Ostrobothnia Hospital District, Oulu, Finland	FinnGen Analysis working group	FinnGen Analysis working group
Katri Pylkäs	University of Oulu, Oulu, Finland	FinnGen Analysis working group	FinnGen Analysis working group
Eeva Sliz	University of Oulu, Oulu, Finland	FinnGen Analysis working group	FinnGen Analysis working group
Minna Karjalainen	University of Oulu, Oulu, Finland	FinnGen Analysis working group	FinnGen Analysis working group
Tuomo Mantere	Northern Finland Biobank Borealis / University of Oulu / Northern Ostrobothnia Hospital District, Oulu, Finland	FinnGen Analysis working group	FinnGen Analysis working group
Eeva Kangasniemi	Finnish Clinical Biobank Tampere / University of Tampere / Pirkanmaa Hospital District, Tampere, Finland	FinnGen Analysis working group	FinnGen Analysis working group
Sami Heikkinen	University of Eastern Finland, Kuopio, Finland	FinnGen Analysis working group	FinnGen Analysis working group
Arto Mannermaa	Biobank of Eastern Finland / University of Eastern Finland / Northern Savo Hospital District, Kuopio, Finland	FinnGen Analysis working group	FinnGen Analysis working group
Eija Laakkonen	University of Jyväskylä, Jyväskylä, Finland	FinnGen Analysis working group	FinnGen Analysis working group
Nina Pitkänen	Auria Biobank / University of Turku / Hospital District of Southwest Finland, Turku, Finland	FinnGen Analysis working group	FinnGen Analysis working group
Samuel Lessard	Translational Sciences, Sanofi R&D, Framingham, MA, USA	FinnGen Analysis working group	FinnGen Analysis working group
Clément Chatelain	Translational Sciences, Sanofi R&D, Framingham, MA, USA	FinnGen Analysis working group	FinnGen Analysis working group
Perttu Terho	Auria Biobank / University of Turku / Hospital District of Southwest Finland, Turku, Finland	Biobank directors	Biobank directors
Tiina Wahlfors	THL Biobank / Finnish Institute for Health and Welfare (THL), Helsinki, Finland	Biobank directors	Biobank directors
Jukka Partanen	Finnish Red Cross Blood Service / Finnish Hematology Registry and Clinical Biobank, Helsinki, Finland	Biobank directors	Biobank directors
Eero Punkka	Helsinki Biobank / Helsinki University and Hospital District of Helsinki and Uusimaa, Helsinki	Biobank directors	Biobank directors
Raisa Serpi	Northern Finland Biobank Borealis / University of Oulu / Northern Ostrobothnia Hospital District, Oulu, Finland	Biobank directors	Biobank directors
Sanna Siltanen	Finnish Clinical Biobank Tampere / University of Tampere / Pirkanmaa Hospital District, Tampere, Finland	Biobank directors	Biobank directors
Veli-Matti Kosma	Biobank of Eastern Finland / University of Eastern Finland / Northern Savo Hospital District, Kuopio, Finland	Biobank directors	Biobank directors
Teijo Kuopio	Central Finland Biobank / University of Jyväskylä / Central Finland Health Care District, Jyväskylä, Finland	Biobank directors	Biobank directors
Anu Jalanko	Institute for Molecular Medicine Finland (FIMM), HiLIFE, University of Helsinki, Helsinki, Finland	FinnGen Teams	Administration
Huei-Yi Shen	Institute for Molecular Medicine Finland (FIMM), HiLIFE, University of Helsinki, Helsinki, Finland	FinnGen Teams	Administration
Risto Kajanne	Institute for Molecular Medicine Finland (FIMM), HiLIFE, University of Helsinki, Helsinki, Finland	FinnGen Teams	Administration
Mervi Aavikko	Institute for Molecular Medicine Finland (FIMM), HiLIFE, University of Helsinki, Helsinki, Finland	FinnGen Teams	Administration
Henna Palin	Finnish Clinical Biobank Tampere / University of Tampere / Pirkanmaa Hospital District, Tampere, Finland	FinnGen Teams	Administration
Malla-Maria Linna	Helsinki Biobank / Helsinki University and Hospital District of Helsinki and Uusimaa, Helsinki	FinnGen Teams	Administration
Juha Karjalainen	Institute for Molecular Medicine Finland (FIMM), HiLIFE, University of Helsinki, Helsinki, Finland	FinnGen Teams	Analysis
Mitja Kurki	Institute for Molecular Medicine Finland (FIMM), HiLIFE, University of Helsinki, Helsinki, Finland	FinnGen Teams	Analysis
Pietro Della Briotta Parolo	Institute for Molecular Medicine Finland (FIMM), HiLIFE, University of Helsinki, Helsinki, Finland	FinnGen Teams	Analysis
Arto Lehisto	Institute for Molecular Medicine Finland (FIMM), HiLIFE, University of Helsinki, Helsinki, Finland	FinnGen Teams	Analysis
Juha Mehtonen	Institute for Molecular Medicine Finland (FIMM), HiLIFE, University of Helsinki, Helsinki, Finland	FinnGen Teams	Analysis
Wei Zhou	Broad Institute, Cambridge, MA, United States	FinnGen Teams	Analysis
Masahiro Kanai	Broad Institute, Cambridge, MA, United States	FinnGen Teams	Analysis
Mutaamba Maasha	Broad Institute, Cambridge, MA, United States	FinnGen Teams	Analysis
Zhili Zheng	Broad Institute, Cambridge, MA, United States	FinnGen Teams	Analysis
Hannele Laivuori	Institute for Molecular Medicine Finland (FIMM), HiLIFE, University of Helsinki, Helsinki, Finland	FinnGen Teams	Clinical Endpoint Development
Aki Havulinna	Institute for Molecular Medicine Finland (FIMM), HiLIFE, University of Helsinki, Helsinki, Finland	FinnGen Teams	Clinical Endpoint Development

Susanna Lemmelä	Institute for Molecular Medicine Finland (FIMM), HiLIFE, University of Helsinki, Helsinki, Finland	FinnGen Teams	Clinical Endpoint Development
Tuomo Kiiskinen	Institute for Molecular Medicine Finland (FIMM), HiLIFE, University of Helsinki, Helsinki, Finland	FinnGen Teams	Clinical Endpoint Development
Elisa Lahtela	Institute for Molecular Medicine Finland (FIMM), HiLIFE, University of Helsinki, Helsinki, Finland	FinnGen Teams	Clinical Endpoint Development
Mari Kaunisto	Institute for Molecular Medicine Finland (FIMM), HiLIFE, University of Helsinki, Helsinki, Finland	FinnGen Teams	Communication
Elina Kilpeläinen	Institute for Molecular Medicine Finland (FIMM), HiLIFE, University of Helsinki, Helsinki, Finland	FinnGen Teams	E-Science
Timo P. Sipilä	Institute for Molecular Medicine Finland (FIMM), HiLIFE, University of Helsinki, Helsinki, Finland	FinnGen Teams	E-Science
Oluwaseun Alexander Dad	Institute for Molecular Medicine Finland (FIMM), HiLIFE, University of Helsinki, Helsinki, Finland	FinnGen Teams	E-Science
Awaisa Ghazal	Institute for Molecular Medicine Finland (FIMM), HiLIFE, University of Helsinki, Helsinki, Finland	FinnGen Teams	E-Science
Anastasia Kytölä	Institute for Molecular Medicine Finland (FIMM), HiLIFE, University of Helsinki, Helsinki, Finland	FinnGen Teams	E-Science
Sanni Ruotsalainen	Institute for Molecular Medicine Finland (FIMM), HiLIFE, University of Helsinki, Helsinki, Finland	FinnGen Teams	E-Science
Rigbe Weldatsadik	Institute for Molecular Medicine Finland (FIMM), HiLIFE, University of Helsinki, Helsinki, Finland	FinnGen Teams	E-Science
Kati Donner	Institute for Molecular Medicine Finland (FIMM), HiLIFE, University of Helsinki, Helsinki, Finland	FinnGen Teams	Genotyping
Timo P. Sipilä	Institute for Molecular Medicine Finland (FIMM), HiLIFE, University of Helsinki, Helsinki, Finland	FinnGen Teams	Genotyping
Anu Loukola	Helsinki Biobank / Helsinki University and Hospital District of Helsinki and Uusimaa, Helsinki	FinnGen Teams	Sample Collection Coordination
Päivi Laiho	THL Biobank / Finnish Institute for Health and Welfare (THL), Helsinki, Finland	FinnGen Teams	Sample Logistics
Tuuli Sistonen	THL Biobank / Finnish Institute for Health and Welfare (THL), Helsinki, Finland	FinnGen Teams	Sample Logistics
Essi Kaiharju	THL Biobank / Finnish Institute for Health and Welfare (THL), Helsinki, Finland	FinnGen Teams	Sample Logistics
Markku Laukkanen	THL Biobank / Finnish Institute for Health and Welfare (THL), Helsinki, Finland	FinnGen Teams	Sample Logistics
Elina Järvensivu	THL Biobank / Finnish Institute for Health and Welfare (THL), Helsinki, Finland	FinnGen Teams	Sample Logistics
Sini Lähteenmäki	THL Biobank / Finnish Institute for Health and Welfare (THL), Helsinki, Finland	FinnGen Teams	Sample Logistics
Lotta Männikkö	THL Biobank / Finnish Institute for Health and Welfare (THL), Helsinki, Finland	FinnGen Teams	Sample Logistics
Regis Wong	THL Biobank / Finnish Institute for Health and Welfare (THL), Helsinki, Finland	FinnGen Teams	Sample Logistics
Auli Toivola	THL Biobank / Finnish Institute for Health and Welfare (THL), Helsinki, Finland	FinnGen Teams	Sample Logistics
Minna Brunfeldt	THL Biobank / Finnish Institute for Health and Welfare (THL), Helsinki, Finland	FinnGen Teams	Registry Data Operations
Hannele Mattsson	THL Biobank / Finnish Institute for Health and Welfare (THL), Helsinki, Finland	FinnGen Teams	Registry Data Operations
Kati Kristiansson	THL Biobank / Finnish Institute for Health and Welfare (THL), Helsinki, Finland	FinnGen Teams	Registry Data Operations
Susanna Lemmelä	Institute for Molecular Medicine Finland (FIMM), HiLIFE, University of Helsinki, Helsinki, Finland	FinnGen Teams	Registry Data Operations
Sami Koskelainen	THL Biobank / Finnish Institute for Health and Welfare (THL), Helsinki, Finland	FinnGen Teams	Registry Data Operations
Tero Hiekkalinna	THL Biobank / Finnish Institute for Health and Welfare (THL), Helsinki, Finland	FinnGen Teams	Registry Data Operations
Teemu Paajanen	THL Biobank / Finnish Institute for Health and Welfare (THL), Helsinki, Finland	FinnGen Teams	Registry Data Operations
Priit Palta	Institute for Molecular Medicine Finland (FIMM), HiLIFE, University of Helsinki, Helsinki, Finland	FinnGen Teams	Sequencing Informatics
Kalle Pärn	Institute for Molecular Medicine Finland (FIMM), HiLIFE, University of Helsinki, Helsinki, Finland	FinnGen Teams	Sequencing Informatics
Mart Kals	Institute for Molecular Medicine Finland (FIMM), HiLIFE, University of Helsinki, Helsinki, Finland	FinnGen Teams	Sequencing Informatics
Shuang Luo	Institute for Molecular Medicine Finland (FIMM), HiLIFE, University of Helsinki, Helsinki, Finland	FinnGen Teams	Sequencing Informatics
Tarja Laitinen	Pirkanmaa Hospital District, Tampere, Finland	FinnGen Teams	Trajectory
Mary Pat Reeve	Institute for Molecular Medicine Finland (FIMM), HiLIFE, University of Helsinki, Helsinki, Finland	FinnGen Teams	Trajectory
Shanmukha Sampath Padsam	Institute for Molecular Medicine Finland (FIMM), HiLIFE, University of Helsinki, Helsinki, Finland	FinnGen Teams	Trajectory
Marianna Niemi	University of Tampere, Tampere, Finland	FinnGen Teams	Trajectory
Harri Siirtola	University of Tampere, Tampere, Finland	FinnGen Teams	Trajectory
Javier Gracia-Tabuenca	University of Tampere, Tampere, Finland	FinnGen Teams	Trajectory
Mika Helminen	University of Tampere, Tampere, Finland	FinnGen Teams	Trajectory
Tiina Luukkaala	University of Tampere, Tampere, Finland	FinnGen Teams	Trajectory
Iida Vähätalo	University of Tampere, Tampere, Finland	FinnGen Teams	Trajectory
Jyrki Tammerluoto	Institute for Molecular Medicine Finland (FIMM), HiLIFE, University of Helsinki, Helsinki, Finland	FinnGen Teams	Data protection officer

Marco Hautalahti	Finnish Biobank Cooperative - FINBB	FinnGen Teams	FINBB - Finnish biobank cooperative
Johanna Mäkelä	Finnish Biobank Cooperative - FINBB	FinnGen Teams	FINBB - Finnish biobank cooperative
Sarah Smith	Finnish Biobank Cooperative - FINBB	FinnGen Teams	FINBB - Finnish biobank cooperative
Tom Southerington	Finnish Biobank Cooperative - FINBB	FinnGen Teams	FINBB - Finnish biobank cooperative
Petri Lehto	Finnish Biobank Cooperative - FINBB	FinnGen Teams	FINBB - Finnish biobank cooperative
