# Supplementary material for: Disentangling the link between maternal influences on birth weight and disease risk in 36,211 genotyped mother–child pairs
Source: Commun Biol. 2024 Feb 12;7:175. doi: 10.1038/s42003-024-05872-9 (PMC10861556; doi:10.1038/s42003-024-05872-9)
Supplement: Supplementary file 3 — Description of Additional Supplementary Files [file 42003_2024_5872_MOESM3_ESM.pdf]

Description of Additional Supplementary Files

File name: Supplementary Data 1
Description: Effect of the six different birth weight PGSs on measured own and child birth weight in FinnGen R10 and parent-child pairs.

File name: Supplementary Data 2
Description: Association of one's own birth weight PGSs with disease risk in FinnGen R10.

File name: Supplementary Data 3
Description: Simulations of true vs. observed maternal effects under different models, combined with estimates of power to detect statistically significant associations with the chosen effect sizes.

File name: Supplementary Data 4
Description: Results from mother-child pairs, regressing maternal PGSs for birth weight with disease risk in the children, with and without adjusting for child/mother genetics (N=36,211).

File name: Supplementary Data 5
Description: Effect of PGS on disease risk in the mother-child pairs using only one child per mother (N=28,792), in strictly unrelated mother-child pairs (N=22,454) and adjusting the original results with a PGS for gestational length (N=36,211).

File name: Supplementary Data 6
Description: Results from father-child pairs, regressing maternal PGSs for birth weight with disease risk in the children, adjusting for paternal/child genetics (N=31,775).

File name: Supplementary Data 7
Description: Results from sibling analyses, estimating the effect of the studied PGSs on disease risk in sibling pairs.

File name: Supplementary Data 8
Description: SNPs for M-SPECIFIC PGS.

File name: Supplementary Data 9
Description: SNPs for M-ALL PGS

File name: Supplementary Data 10
Description: SNPs for MF-ALL PGS

File name: Supplementary Data 11
Description: SNPs for F-SPECIFIC PGS
