# Supplementary material for: Disentangling the link between maternal influences on birth weight and disease risk in 36,211 genotyped mother–child pairs
Source: Commun Biol. 2024 Feb 12;7:175. doi: 10.1038/s42003-024-05872-9 (PMC10861556; doi:10.1038/s42003-024-05872-9)
Supplement: Supplementary file 5 — Reporting Summary [file 42003_2024_5872_MOESM5_ESM.pdf]

nature portfolio

Corresponding author(s): Taru Tukiainen

Last updated by author(s): Jan 18, 2024

Reporting Summary

Nature Portfolio wishes to improve the reproducibility of the work that we publish. This form provides structure for consistency and transparency in reporting. For further information on Nature Portfolio policies, see our Editorial Policies and the Editorial Policy Checklist.

Statistics

For all statistical analyses, confirm that the following items are present in the figure legend, table legend, main text, or Methods section.

n/a	Confirmed	
☐	☒	The exact sample size (*n*) for each experimental group/condition, given as a discrete number and unit of measurement
☒	☐	A statement on whether measurements were taken from distinct samples or whether the same sample was measured repeatedly
☐	☒	The statistical test(s) used AND whether they are one- or two-sided<br>*Only common tests should be described solely by name; describe more complex techniques in the Methods section.*
☐	☒	A description of all covariates tested
☐	☒	A description of any assumptions or corrections, such as tests of normality and adjustment for multiple comparisons
☐	☒	A full description of the statistical parameters including central tendency (e.g. means) or other basic estimates (e.g. regression coefficient) AND variation (e.g. standard deviation) or associated estimates of uncertainty (e.g. confidence intervals)
☐	☒	For null hypothesis testing, the test statistic (e.g. *F*, *t*, *r*) with confidence intervals, effect sizes, degrees of freedom and *P* value noted<br>*Give P values as exact values whenever suitable.*
☒	☐	For Bayesian analysis, information on the choice of priors and Markov chain Monte Carlo settings
☒	☐	For hierarchical and complex designs, identification of the appropriate level for tests and full reporting of outcomes
☐	☒	Estimates of effect sizes (e.g. Cohen's *d*, Pearson's *r*), indicating how they were calculated

Our web collection on statistics for biologists contains articles on many of the points above.

Software and code

Policy information about availability of computer code

Data collection	No software was used for data collection.
Data analysis	Description of power analyses used in the study is available as supplementary code.<br>The full genotyping and imputation protocol for FinnGen is described at https://doi.org/10.17504/protocols.io.nmndc5e.<br>We utilized the following software in our analyses:<br>KING (https://www.kingrelatedness.com/) - identification of parent-offspring relationships.<br>plink2 (www.cog-genomics.org/plink/2.0/) - calculation of lead SNP based genetic scores.<br>PRS-CS (https://github.com/getian107/PRScs) - calculation of genome-wide genetic scores.<br>Statistical analyses were performed using R Statistical Software (v4.3; R Core Team 2023).

For manuscripts utilizing custom algorithms or software that are central to the research but not yet described in published literature, software must be made available to editors and reviewers. We strongly encourage code deposition in a community repository (e.g. GitHub). See the Nature Portfolio guidelines for submitting code & software for further information.

Data

Policy information about availability of data

All manuscripts must include a data availability statement. This statement should provide the following information, where applicable:

- Accession codes, unique identifiers, or web links for publicly available datasets
- A description of any restrictions on data availability
- For clinical datasets or third party data, please ensure that the statement adheres to our policy

Full genetic and clinical data from FinnGen is available for researchers by application (https://www.finngen.fi/en/access_results). Details of FinnGen core endpoints can be found at risteys.finngen.fi. The source data for results figures is available in Supplementary Data: for Fig. 2 – Supplementary Data 4; Supplementary Fig. 2 – Supplementary Data 3; Supplementary Fig. 3 – Supplementary Data 4; Supplementary Fig. 4 – Supplementary Data 4, 5 and 6; Supplementary Fig. 5 – Supplementary Data 6; Supplementary Fig. 6 – Supplementary Data 7.

Research involving human participants, their data, or biological material

Policy information about studies with human participants or human data. See also policy information about sex, gender (identity/presentation), and sexual orientation and race, ethnicity and racism.

Reporting on sex and gender	We divided the identified parent-offspring pairs in the FinnGen release 10 into mother-child and father-child pairs. FinnGen samples with ambiguous sex, e.g., mismatch between reported and genotype-inferred sex were removed during sample-level quality control. We did not stratify the main analyses with child sex.
Reporting on race, ethnicity, or other socially relevant groupings	FinnGen participants included in the study consist of FinnGen samples identified as Finns in genetic principal components analysis (PCA).
Population characteristics	The study included participants from the 10th data relase of FinnGen. Participants included in the main analyses were of Finnish ancestry. After data pruning and quality control, the release 10 of the dataset contains phenotypes for 412,176 participants (181,869 men and 230,307 women, with a median age at the end of followup of 62.9 yrs. We identified 67,986 parent-child relationships within the dataset (36,211 mother-child pairs and 31,775 father child pairs). The mean age of the mothers is 70.0 yrs, the mean age of the fathers 71.2 yrs, and the mean age of the children 46.3 yrs, at the end of followup period.
Recruitment	The FinnGen study (https://www.finngen.fi/en) is an on-going research project that utilizes samples from a nationwide network of Finnish biobanks and digital health care data from national health registers. FinnGen consists of a random sample of subjects from Finnish population-based cohorts and clinical biobanks. The majority of the samples have been gathered from six university hospital biobanks.
Ethics oversight	All patients and control participants in FinnGen provided informed consent for biobank research, based on the Finnish Biobank Act. Research cohorts collected prior the start of FinnGen (in August 2017), were collected based on study-specific consents and later transferred to the Finnish biobanks after approval by Valvira, the National Supervisory Authority for Welfare and Health. Recruitment protocols followed the biobank protocols approved by Valvira.<br>The Coordinating Ethics Committee of the Hospital District of Helsinki and Uusimaa (HUS) approved the FinnGen study protocol Nr HUS/990/2017.<br>The FinnGen study is approved by Finnish Institute for Health and Welfare (THL), approval number THL/2031/6.02.00/2017, amendments THL/1101/5.05.00/2017, THL/341/6.02.00/2018, THL/2222/6.02.00/2018, THL/283/6.02.00/2019, THL/1721/5.05.00/2019, Digital and population data service agency VRK43431/2017-3, VRK/6909/2018-3, VRK/4415/2019-3 the Social Insurance Institution (KELA) KELA 58/522/2017, KELA 131/522/2018, KELA 70/522/2019, KELA 98/522/2019, and Statistics Finland TK-53-1041-17. The Biobank Access Decisions for FinnGen samples and data utilized in FinnGen Data Freeze 10 include: THL Biobank BB2017_55, BB2017_111, BB2018_19, BB_2018_34, BB_2018_67, BB2018_71, BB2019_7, BB2019_8, BB2019_26, BB2020_1, BB2021_65, Finnish Red Cross Blood Service Biobank 7.12.2017, Helsinki Biobank HUS/359/2017, HUS/248/2020, HUS/150/2022 §12, §13, §14, §15, §16, §17, §18, and §23, Auria Biobank AB17-5154 and amendment #1 (August 17 2020) and amendments BB_2021-0140, BB_2021-0156 (August 26 2021, Feb 2 2022), BB_2021-0169, BB_2021-0179, BB_2021-0161, AB20-5926 and amendment #1 (April 23 2020)and it's modification (Sep 22 2021), Biobank Borealis of Northern Finland_2017_1013, 2021_5010, 2021_5018, 2021_5015, 2021_5023, 2021_5017, 2022_6001, Biobank of Eastern Finland 1186/2018 and amendment 22§/2020, 53§/2021, 13§/2022, 14§/2022, 15§/2022, Finnish Clinical Biobank Tampere MH0004 and amendments (21.02.2020 & 06.10.2020), §8/2021, §9/2022, §10/2022, §12/2022, §20/2022, §21/2022, §22/2022, §23/2022, Central Finland Biobank 1-2017, and Terveystalo Biobank STB 2018001 and amendment 25th Aug 2020, Finnish Hematological Registry and Clinical Biobank decision 18th June 2021, Arctic biobank P0844: ARC_2021_1001.

Note that full information on the approval of the study protocol must also be provided in the manuscript.

Field-specific reporting

Please select the one below that is the best fit for your research. If you are not sure, read the appropriate sections before making your selection.

☒ Life sciences ☐ Behavioural & social sciences ☐ Ecological, evolutionary & environmental sciences

Life sciences study design

All studies must disclose on these points even when the disclosure is negative.

Sample size	The study is based on FinnGen data freeze 10 (N=430,897). After sample pruning and quality control we were left with 412,176 FinnGen subjects, containing 36,211 mother-child-pairs, 31,775 father-child pairs and 63,428 full sibling pairs. In the main analyses in mother-child pairs, the number of children with a confirmed disease endpoint ranged from 996 CHD cases to 6,150 statin users. More details of the sample size for each analyses are available in the manuscript and in the supplementary data.
Data exclusions	Exclusion of samples and variants was based on standard guidelines and quality control procedures and are described in the Methods of the manuscript. The exclusion criteria were pre-established.
Replication	We used several sensitivity analyses to corroborate our main findings. These include using two stringently filtered datasets of mother-child pairs, father-child pairs and a distinct dataset of FinnGen siblings. Details of each dataset are available in the manuscript.
Randomization	The study does not include allocation of paticipants to different experimental groups.
Blinding	No blinding was necessary for this observational study.

Reporting for specific materials, systems and methods

We require information from authors about some types of materials, experimental systems and methods used in many studies. Here, indicate whether each material, system or method listed is relevant to your study. If you are not sure if a list item applies to your research, read the appropriate section before selecting a response.

Materials & experimental systems

n/a	Involved in the study
☒	☐ Antibodies
☒	☐ Eukaryotic cell lines
☒	☐ Palaeontology and archaeology
☒	☐ Animals and other organisms
☒	☐ Clinical data
☒	☐ Dual use research of concern
☒	☐ Plants

Methods

n/a	Involved in the study
☒	☐ ChIP-seq
☒	☐ Flow cytometry
☒	☐ MRI-based neuroimaging

Plants

Seed stocks	*Report on the source of all seed stocks or other plant material used. If applicable, state the seed stock centre and catalogue number. If plant specimens were collected from the field, describe the collection location, date and sampling procedures.*
Novel plant genotypes	*Describe the methods by which all novel plant genotypes were produced. This includes those generated by transgenic approaches, gene editing, chemical/radiation-based mutagenesis and hybridization. For transgenic lines, describe the transformation method, the number of independent lines analyzed and the generation upon which experiments were performed. For gene-edited lines, describe the editor used, the endogenous sequence targeted for editing, the targeting guide RNA sequence (if applicable) and how the editor was applied.*
Authentication	*Describe any authentication procedures for each seed stock used or novel genotype generated. Describe any experiments used to assess the effect of a mutation and, where applicable, how potential secondary effects (e.g. second site T-DNA insertions, mosiacism, off-target gene editing) were examined.*
